# An Overview of Liquid Chromatography–Mass Spectrometry (LC–MS) Methods for the Quantification of Antibody‐Drug Conjugates

**DOI:** 10.1002/bmc.70334

**Published:** 2026-01-07

**Authors:** Pauline L. M. Buitelaar, Hilde Rosing, Jos H. Beijnen, Neeltje Steeghs, Alwin D. R. Huitema

**Affiliations:** ^1^ Department of Pharmacy & Pharmacology Antoni van Leeuwenhoek Hospital/the Netherlands Cancer Institute Amsterdam the Netherlands; ^2^ Department of Clinical Pharmacology, Division of Medical Oncology the Netherlands Cancer Institute Amsterdam the Netherlands; ^3^ Department of Medical Oncology University Medical Centre Utrecht, Utrecht University Utrecht the Netherlands; ^4^ Department of Clinical Pharmacy University Medical Center Utrecht, Utrecht University Utrecht the Netherlands; ^5^ Department of Pharmacology Princess Máxima Center for Pediatric Oncology Utrecht the Netherlands

**Keywords:** antibody‐drug conjugates, bioanalysis, LC–MS, literature review

## Abstract

Antibody‐drug conjugates (ADCs) are innovative drugs composed of cytotoxic molecules (payload) linked to antibodies, that selectively target and kill cancer cells upon internalization. In vivo, ADCs exist as intact molecules, naked antibodies, or released, unconjugated (linker‐)payload. Accurate quantification of these entities is crucial for understanding ADCs pharmacokinetics. Ligand‐binding assays are commonly used to measure ADC concentrations and total antibody concentrations, whereas LC–MS/MS is used to analyze the payload. Due to limitations in ligand‐binding assays, this review focuses on quantitative LC–MS methods for the different ADC entities. Quantitative LC–MS assays were described for all ADC entities, available from full manuscripts and regulatory reviews of 12 ADCs evaluated by the European Medicine Agency, by January 2025. The review summarized sample pre‐treatment, chromatography, mass spectrometry, validation, and stability data for each LC–MS method. Overall, critical details were often missing, particularly concerning sample pre‐treatment, validation criteria, and sample stability. In conclusion, LC–MS quantification of ADC entities is feasible but current methods lack sufficient detail. Our review highlights the need for further research to develop reliable LC–MS assays for ADCs. This review may serve as a starting point and outlines key factors to consider in future LC–MS method development.

## 1 Introduction

Antibody‐drug conjugates (ADCs) are a new class of drugs based on the “magic bullet” principle proposed by Paul Ehrlich (Strebhardt and Ullrich 2008). ADCs consist of cytotoxic molecules that are attached to a monoclonal antibody through a linker. The monoclonal antibody specifically binds to antigens expressed on cancer cells, thereby facilitating targeted delivery of the cytotoxic molecules to malignant cells and inducing cancer‐specific cytotoxicity. In this way, unintended delivery of the cytotoxic agent to healthy tissue could be minimized, thereby increasing drug tolerability (Marei et al. 2022; Strebhardt and Ullrich 2008). Despite the long history of the “magic bullet” principle, the development of ADCs has only recently accelerated with advances in monoclonal antibody engineering and success of antibody‐based drugs, e.g., rituximab, trastuzumab, and immunotherapy drugs (Shen 2015). The first ADC, gemtuzumab ozogamicin (Mylotarg), received regulatory approval from the European Medicines Agency (EMA) and U.S. Food and Drug Administration (FDA) for the treatment of acute myeloid leukemia in 2000 (*European Medicines Agency. Mylotarg*; *FDA U.S. Food & Drug Administration. Drugs@FDA. Drug Approval Package—Mylotarg (gemtuzumab ozogamicin) Injection*). Currently, 10 ADCs have been authorized in Europe, two are under regulatory review, and over 100 investigational ADCs are in various stages of clinical trials globally (Shastry et al. 2023).

With the increasing clinical use of ADCs, more information on their clinical pharmacology has become available. Emerging evidence suggests that ADC efficacy may involve mechanisms beyond the “magic bullet” principle. It has been reported that less than 1% of the ADC concentration reaches the tumor site, mainly due to the limited tissue penetration and accumulation of the large ADC molecule (Colombo and Rich 2022). Although low ADC concentrations reach the tumor, these may still be sufficient to induce tumor cell death. Notably, ADCs have demonstrated antitumor activity even in tumors with low or negative antigen expression (Colombo and Rich 2022), indicating that therapeutic efficacy is not solely dependent on antigen binding. Additionally, observed adverse events with ADC treatment vary and could be off‐ or on‐site and off‐ or on‐target (Nguyen et al. 2023), which may reflect premature systemic release of the cytotoxic molecule. Moreover, the linker‐type and antibody component could also contribute to efficacy and toxicity (Vogel et al. 2025).

**FIGURE 1:**
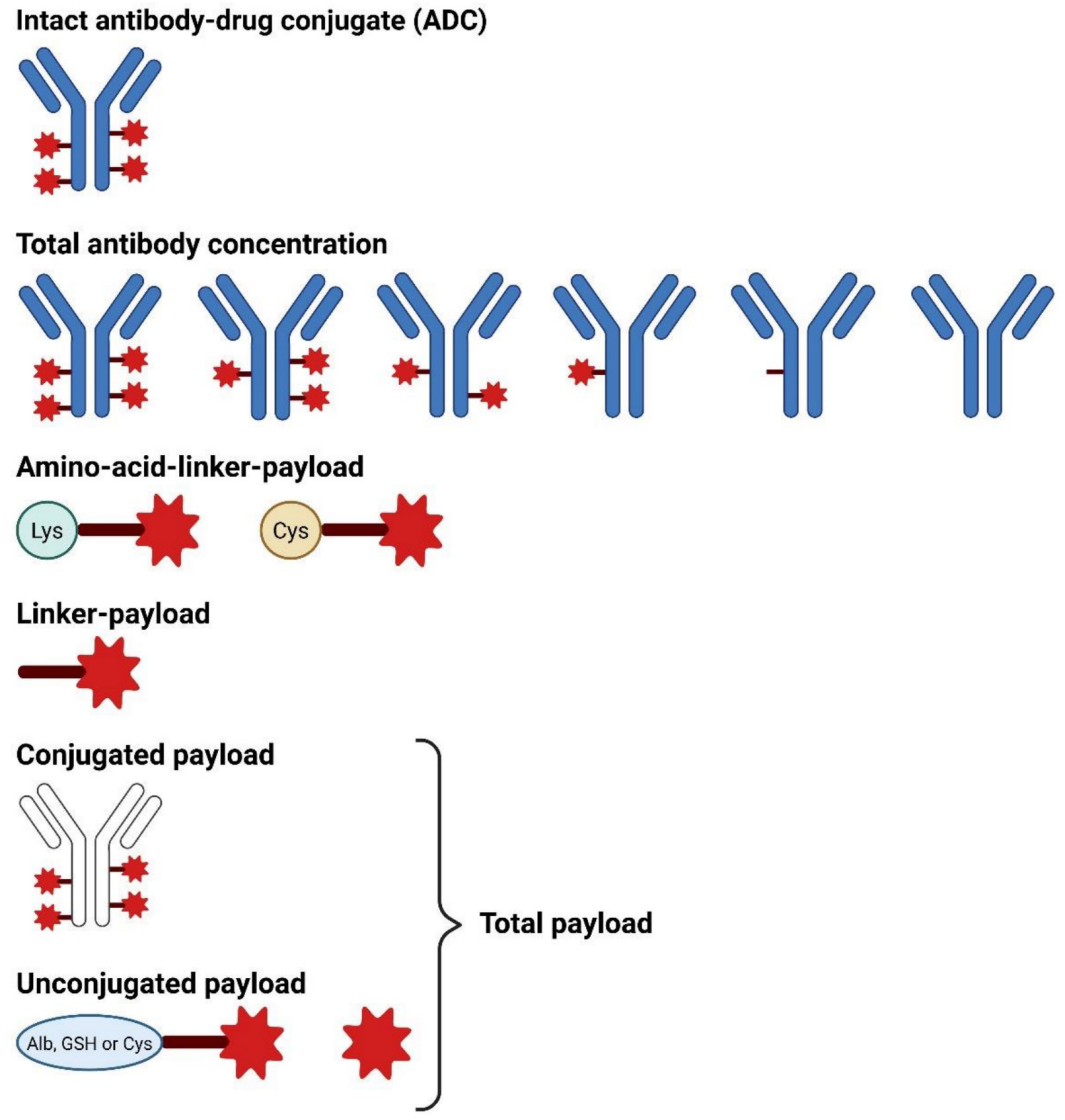
The general structure of an antibody‐drug conjugate. The monoclonal antibody is depicted in black, to which via a linker (dark red line) the cytotoxic molecule, or payload, (red star) is attached. Image was created with BioRender.

Given these observations, pharmacokinetic research is needed to further study the clinical pharmacology of ADCs and establish possible concentration‐effect and concentration‐toxicity relationships. Based on the structure of an ADC, displayed in Figure 1, multiple entities can be distinguished and measured. First, the intact ADC can, at least theoretically, be measured. Second, the total antibody concentration, often used as a proxy for the total ADC concentration, can be assessed. Third, the concentration of the cytotoxic molecule in the systemic circulation, also known as free payload, can be measured. Lastly, total payload can be determined after forced release of cytotoxic payload. Two types of bioanalytical methods are generally used to measure these ADC entities. Intact ADC and total antibody concentrations are most frequently measured with ligand binding assays, such as ELISA, whereas payload with or without linker concentrations are measured with LC–MS/MS (Qin and Gong 2022).

Measurement of intact ADC and total antibody concentrations with ELISA presents challenges compared to LC–MS‐based methods. Accurate quantification of intact ADC concentrations with ELISA is primarily complicated by variability in the drug‐to‐antibody ratio (DAR). Firstly, the recovery of ADCs in traditional sandwich ELISAs is influenced by the drug load, with semi‐homogenous ELISAs being less affected (Kozak et al. 2013). Secondly, accurate measurements of the intact ADC concentration require careful selection of both the capture antibody and detection reagents for differentiation between different DARs. Thirdly, ADCs with varying DAR values perform differently, resulting in inaccurate measurement of very low or very high DARs due to low avidity or steric hindrance (Gorovits et al. 2013). Consequently, in vivo changes in DAR can result in under‐ or overestimation of ADC concentrations (Stephan et al. 2008). Addressing the impact of DAR necessitates the availability of ADCs with distinct DAR profiles, further complicating the development and validation of ELISA‐based methods (Gorovits et al. 2013).

Measurement of the intact ADC and total antibody concentrations with LC–MS represents an alternative to ligand binding assays. LC–high‐resolution mass spectrometry (LC‐HR‐MS) enables direct measurement of the DAR of drug products (Zhu et al. 2020). Moreover, several LC–MS/MS methods for the quantification of therapeutic monoclonal antibodies have already been published (de Jong et al. 2022; Li et al. 2024; Schokker et al. 2020). When adapted for ADCs, these methods have the potential to overcome many limitations of ligand‐binding based assays. Therefore, we suggest that LC–MS‐based techniques would be a favorable bioanalytical technique for the quantification of intact ADC, total antibody, free, and conjugated payload concentrations.

To date, several reviews have been published outlining possible bioanalytical methods or strategies for the determination of intact ADC, total antibody, free, and conjugated payload concentrations (Gorovits et al. 2013; Qin and Gong 2022; Todoroki et al. 2020). However, none have specifically focused on LC–MS‐based approaches for measuring these entities. Therefore, in this review, we focused on published LC–MS methods quantifying different ADC entities of all ADCs approved by the EMA. To facilitate accurate interpretation of the presented data, we also provide background information on the ADC pharmacology and structure.

## 2 Background

### 2.1 Pharmacology of ADCs

#### 2.1.1 Concept of ADCs

**FIGURE 2:**
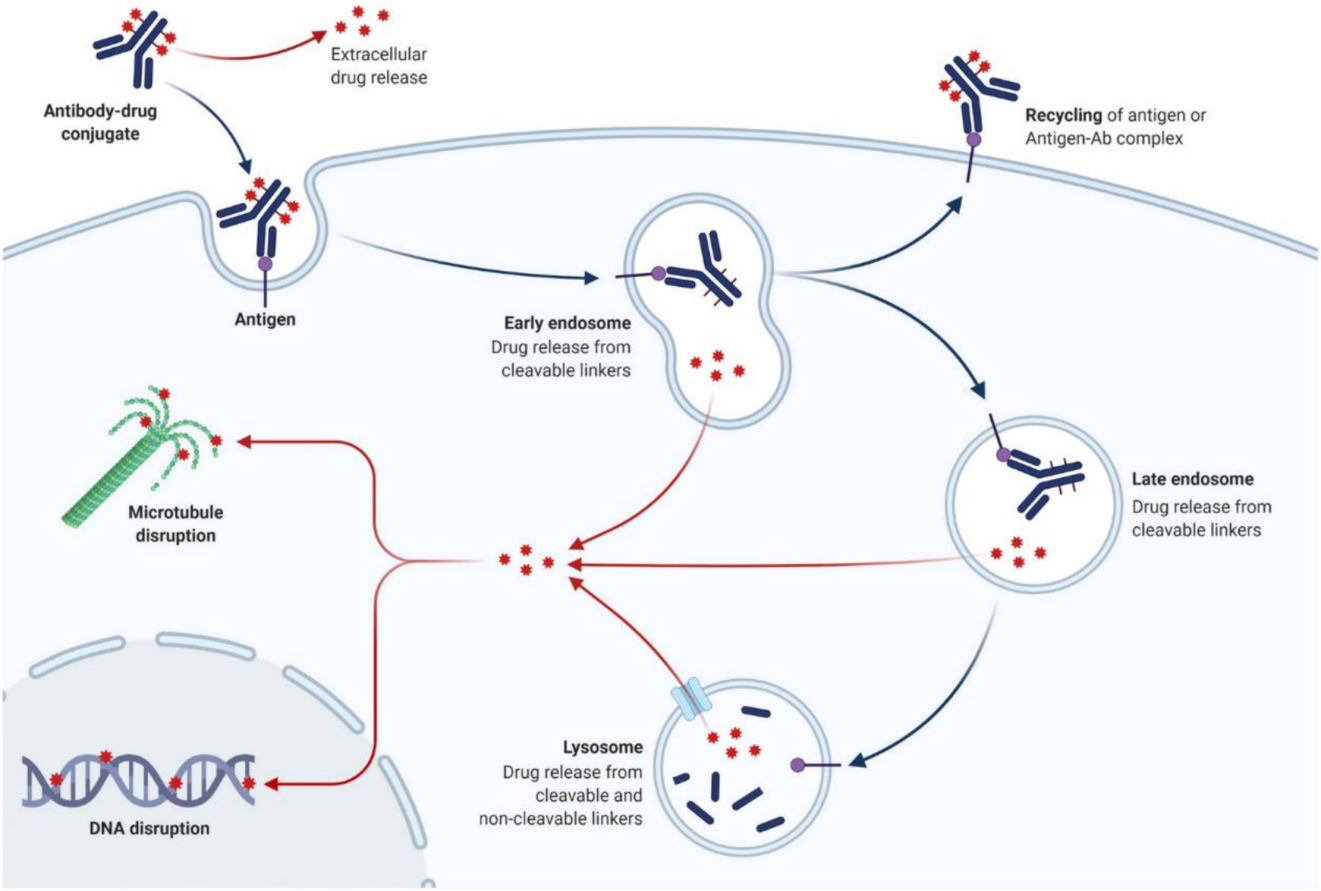
The internalization with cytotoxin release and recycling of antibody‐drug conjugates. Figure was created with BioRender and published by Marei et al. (2022).

The aim of an ADC is specific delivery of a cytotoxic agent to a tumor cell without unintended delivery of cytotoxic payload to healthy tissues. Figure 2 provides a schematic overview of the ADC's targeting, internalization, and intracellular mechanisms. The antibody moiety of the ADC binds to a specific target antigen present on a tumor cell. Subsequently, the ADC‐antigen complex is internalized predominantly via clathrin‐mediated endocytosis, resulting in the formation of ADC‐containing endosomes. Within the acidic endosomal environment, interaction between the antibody moiety and the neonatal fragment crystallizable receptor (FcRn) takes place, responsible for recycling of the IgGs as well as IgG‐type ADCs. Moreover, the acidic environment in endosomes facilitates payload release of ADCs with an acid‐sensitive cleavable linker. “Late” endosomes subsequently fuse with lysosomes, with an even lower pH and proteases, such as cathepsin‐B and plasmin. Here, both acid‐sensitive linkers and protease‐sensitive linkers are cleaved, resulting in payload release. ADCs with non‐cleavable linkers rely on lysosomal proteolysis of the antibody to release the cytotoxic agent. Once released, the cytotoxic payload diffuses intracellularly and exerts its effect by targeting DNA, microtubules, or topo‐isomerase I ultimately inducing cell death. Additionally, membrane‐permeable cytotoxic molecules may diffuse out of the targeted, causing cytotoxic effects in neighboring cells, which is called “bystander effect” (Drago et al. 2021; Dumontet et al. 2023; Khongorzul et al. 2020).

#### 2.1.2 Additional Mechanisms of Action of ADCs

The antibody moiety of ADCs could induce other pharmacologic effects as well. First, antigen binding can exert a direct effect (Fu et al. 2022). For example, trastuzumab‐based ADCs bind to HER2, thereby inhibiting dimerization of HER2 with HER1, HER2, or HER3 and subsequent downstream signaling. Second, the antibody entity could engage immune effector mechanisms directed against tumor cells, including complement‐dependent cytotoxicity (CDC), antibody‐dependent cell cytotoxicity (ADCC), and antibody‐dependent cellular phagocytosis (ADCP) (Fu et al. 2022). Activation of the complement cascade, natural killer cells, and macrophages will cause cancer cell death. All ADCs utilize immunoglobulin subtype G (IgGs) subtypes as their antibody backbone, with IgG1 and IgG3 exhibiting the highest potency in mediating immune effector functions, whereas IgG2 and IgG4 subtypes induce these effects to a lesser extent (Vidarsson et al. 2014).

#### 2.1.3 Pharmacokinetics of the Cytotoxic Payload

The ultimate goal of ADCs is to expose tumor cells to cytotoxic payload. Conjugation of a cytotoxic molecule to an antibody alters the pharmacokinetic profile of the payload substantially. The half‐life of the cytotoxic molecule is prolonged due to IgG recycling via the FcRn‐pathway and their protection from clearance by the kidneys (Colombo and Rich 2022). Given that FcRn‐recycling occurs in acidic lysosomes, ADCs containing pH‐sensitive linkers may encounter stability challenges under these conditions (Lencer and Blumberg 2005). Furthermore, the pharmacokinetics of the released payload can be influenced by the conjugation chemistry, for example in cysteine‐conjugated ADCs. The thioether (C–S) bond connecting the linker‐payload to the monoclonal antibody is susceptible to cleavage through Michael addition reactions (Gupta et al. 2017). The resulting linker‐payload intermediate is capable of reacting with other thiol‐containing molecules such as serum albumin (Colombo et al. 2024; Gupta et al. 2017). Binding of linker‐payload to albumin extends its half‐life due to albumin recycling via the FcRn‐pathway (Colombo and Rich 2022). Notably, the albumin–linker‐payload complex may be internalized by tumor cells, where its catabolism can result in payload release, potentially enhancing antitumor efficacy. However, the prolonged systemic exposure and non‐specific distribution of albumin‐bound payloads may also increase the risk of off‐target toxicity.

### 2.2 Structure of ADCs

The general structure of an ADC is an antigen‐specific monoclonal antibody to which a cytotoxic molecule is coupled via a linker (Khongorzul et al. 2020). The general structure of an ADC is displayed in Figure 1.

#### 2.2.1 Monoclonal Antibodies

The monoclonal antibody targets a specific antigen, which is predominantly or exclusively expressed on cancer cells with minimal or no expression on healthy tissues (Beck et al. 2017). Effective delivery of the cytotoxic payload requires not only high‐affinity binding of the antibody to the target antigen but also internalization of the ADC‐antigen complex into the tumor cell (Donaghy 2016). However, some studies suggest that internalization may not be an absolute requirement, as early extracellular release of the payload in the tumor micro‐environment can mediate bystander killing of neighboring cancer cells (Casi and Neri 2015; Staudacher and Brown 2017).

The monoclonal antibodies used for ADCs are typically of IgG1 or IgG4 subtype (Fu et al. 2022). Differences in IgG subtype result in differences in FcγR‐binding efficiency, immune effector activation, overall immunogenicity, and half‐life, which are most favorable for the IgG1 subtype (Fu et al. 2022; Tsuchikama et al. 2024; Vidarsson et al. 2014). Additionally, antibody effector functions can be modulated by post‐translational modifications, such as glycosylation. IgG1 antibodies contain an N‐linked glycan at the asparagine 297 residue (Asn^297^) on each heavy chains. This N‐linked glycan is formed by the addition of various sugar moieties resulting in different glycoforms (Zheng et al. 2011). Five major glycoforms have been identified, distinguished by the presence or absence of galactose or fucose residues: G0/G0F, G0F/G0F, G0F/G1F, G1F/G1F (isomeric with G0F/G2F), and G1F/G2F (Senini et al. 2024).

#### 2.2.2 Linkers

Linkers serve as bridge between the monoclonal antibody and the cytotoxic payload. In approved ADCs, the linker‐payload moiety is typically conjugated to either lysines or cysteines of the monoclonal antibody (Tsuchikama and An 2018).

Lysine‐based conjugation is achieved via amide bond formation, whereby the activated carboxylic acid group of the linker reacts with the ε‐amino group of lysine residues. Conjugation to lysines is heterogeneous due to the widespread distribution of lysines on the antibody surface. Approximately 10 out of the 80 lysine residues are accessible for conjugation, generally resulting in an average DAR of three to four for lysine conjugated ADCs (Tsuchikama and An 2018).

Cysteine‐based conjugated is facilitated by the selective reduction of interchain disulfide bonds, which are non‐essential for IgG1 stability. This reduction exposes the free thiol groups that react with thiol‐reactive groups in the linker. Given that IgG1 molecules contain four interchain disulfide bonds, a maximum of eight free thiols at predetermined places in the monoclonal antibody can participate in conjugation. This leads to less heterogeneity and a more controlled DAR compared to lysine conjugation (Tsuchikama and An 2018).

The physicochemical properties of the linker are critical for ADC performance. Linker stability influences premature payload release in the systemic circulation, potentially affecting therapeutic efficacy and safety (Khongorzul et al. 2020). Furthermore, high hydrophobicity of the linker could promote ADC aggregation, increasing the risk of hepatotoxicity or immunogenicity (Tsuchikama and An 2018). To mitigate these effects, hydrophilic moieties, such as polyethylene glycol (PEG) chains are sometimes incorporated in the linker structure.

Broadly, linkers can be categorized as cleavable or non‐cleavable. Linker types that are used in clinically approved ADCs, are described below.

Cleavable linkers contain chemical groups that are susceptible to cleavage under specific physiological conditions or by certain enzymes (Khongorzul et al. 2020). These linkers can be classified into chemically cleavable and enzymatically cleavable.

Chemically cleavable linkers include acid‐labile and reducible linkers. **Acid‐labile linkers** undergo hydrolysis in acidic environments, such as endosomes and lysosomes where the pH ranges from 4 to 6, resulting in cleavage of chemical groups like hydrazones and subsequent payload release. Despite the neutral pH in the systemic circulation, premature payload release has been reported for ADCs containing acid‐labile linkers, including gemtuzumab ozogamicin, inotuzumab ozogamicin, and sacituzumab govitecan (Khongorzul et al. 2020; Su et al. 2021; Tsuchikama and An 2018). This instability is reflected by relatively short plasma half‐lives of these ADCs, ranging from 36 to 48 hours (Khongorzul et al. 2020).

**Reducible linkers** typically contain disulfide or thio‐ether bonds that are cleaved primarily through reduction by intracellular glutathione, which is present at millimolar concentrations intracellularly (1–10 mM) compared to micromolar concentrations extracellularly and in plasma (2–50 μM) (Griffith 1999; Sheyi et al. 2022). Elevated intracellular glutathione levels in cancer cells, associated with cell survival, tumor growth, and cell stress conditions in hypoxia (Khongorzul et al. 2020), facilitate a more selective cleavage of reducible linkers within the tumor micro‐environment than acid‐cleavable linkers. Consequently, reducible linkers exhibit enhanced stability in the circulation relative to acid‐labile linkers, thereby minimizing premature payload release. The released payloads are generally neutrally charged, promoting efficient cell membrane permeability and facilitating bystander killing (Drago et al. 2021).

Enzymatically cleavable linkers are often engineered with self‐immolative spacers, such as *para*‐aminobenzyl (PAB) groups, which provide steric accessibility required for enzymatic cleavage (Su et al. 2021). These spacers undergo spontaneous 1,6‐elimination following enzymatic cleavage, resulting in rapid and selective release of the payload. **Protease‐sensitive linkers** are composed of multiple peptides that are cleaved by lysosomal proteases, for example cathepsins, which are often overexpressed in tumor cells (Eriksson and Öllinger 2024; Iulianna et al. 2022). Amino acids that are commonly used are valine, either in combination with citrulline or alanine (Khongorzul et al. 2020). Other amino acids could be used as well, such as a combination of glycines and phenylalanines that form a tetrapeptide in trastuzumab deruxtecan (*U.S. Food & Drug Administration (FDA). Highlights of prescribing information—ENHERTU*). Cleavage of these peptide bonds predominantly occurs within lysosomes, enhancing tumor selectivity of payload release. Additionally, cathepsins released from necrotic cells may diffuse into the tumor microenvironment, facilitating extracellular payload release prior to ADC internalization (Drago et al. 2021). In the systemic circulation, payload release is minimal due to suboptimal pH and the presence of serum protease inhibitors (Sheyi et al. 2022).

Unlike cleavable linkers, non‐cleavable linkers lack reactive chemical bonds that can be selectively cleaved under specific physiological conditions or by specific enzymes. The release of the payload in case of non‐cleavable linkers primarily depends on lysosomal proteolytic degradation of the antibody. As a result, a residual amino acid could be covalently attached to a linker‐payload or, following amino acid hydrolysis, the linker‐payload moiety alone. The presence of the attached linker and/or amino acid may modulate both the potency and membrane permeability of the cytotoxic molecule, thereby influencing the extent of bystander killing (McCombs and Owen 2015). For two ADCs, trastuzumab emtansine and belantamab mafodotin, the potency of the cytotoxic payloads, DM1 and MMAF, however, was not impaired despite the attachment of the amino acid‐linker moiety (Hunter et al. 2020; McCombs and Owen 2015). Non‐cleavable linkers include often non‐reducible thioether or maleimidocaproyl (mc) functional groups. The non‐reducible properties of these groups increase the stability of the ADC in the systemic circulation compared to ADCs with cleavable linkers (Sheyi et al. 2022).

#### 2.2.3 Cytotoxins

The cytotoxic molecules, also referred to as payload or cytotoxins, are the active components of the ADCs responsible for inducing cell death following targeted delivery. These cytotoxins are typically derived from naturally occurring compounds and can be classified into several distinct groups.

**Calicheamicin** is a natural antitumor antibiotic, which can be isolated from the actinomycete *Micromonospora echinospora* . From this natural component, a semisynthetic derivative *N*‐acetyl‐γ‐calicheamicin 1,2‐dimethylhydrazine was developed and is used as ADC payload. Upon intracellular release, this molecule undergoes a series of chemical transformations. Initially, glutathione reduces the disulfide bridge, yielding a nucleophilic sulfur that initiates an intramolecular Michael reaction. This reaction triggers an intramolecular rearrangement which results in Bergman cyclization, forming a highly reactive diradical (Kraka et al. 2007). This molecule interacts with hydrogens in the minor DNA groove of the DNA double helix, causing double‐stranded DNA breaks. After this interaction, the stable *N*‐acetyl‐ε‐calicheamicin 1,2‐dimethylhydrazine is formed which is inactive and non‐toxic (Tan 2015).

**Pyrrolobenzodiazepines** are natural antitumor agents produced by actinomycetes. In ADCs, pyrrolobenzodiazepine dimers are used as payload. These dimers exert cytotoxicity through covalent binding between the C2‐amino group of a guanine base and the electrophilic N10–C11 imine of the pyrrolobenzodiazepine. Guanines located within the sequence purine‐guanine‐purine are mostly attacked. As a result, DNA alkylation and the formation of cross‐links lead to apoptosis of the cell (Obaji et al. 2024).

Dolastatin 10 is a naturally occurring **auristatin**, which can be isolated from the *Dolabella auricularia* . Synthetic analogs of this molecule, monomethyl auristatin‐E (MMAE) and monomethyl auristatin‐F (MMAF), are used as ADC payloads. Both molecules consist of five amino acids; however, MMAF contains a phenylalanine moiety that contributes to membrane impermeability (Sokka et al. 2019; Tan 2015). Auristatins disrupt microtubule function by the inhibition of microtubule polymerization and tubulin‐dependent GTP hydrolysis, ultimately leading to cell arrest and apoptosis (Tan 2015).

Maytansine was originally isolated from the bark of the African shrub *Maytenus serrata* and *Maytenus buchananii* . This was the first drug in the class of **maytansinoids**, which are ansa macrolide antibiotics. Structural modifications to maytansine have resulted in the formation of maytansine analogs, DM1 and DM4, containing disulfide or thiol groups, suitable for conjugation and use as ADC payloads. Both compounds inhibit tubulin polymerization, cause cell cycle arrest at G2/M‐phase, and inhibit mitosis (Tan 2015). In case of DM4, an uncharged, lipophilic metabolite (S‐methyl‐DM4) is formed, which is very potent in cell toxicity and could diffuse across cell membrane to exert bystander killing (Erickson et al. 2006).

**Camptothecin**, an alkaloid originally isolated from the *Camptotheca acuminate* , served as basis for the development and approval of derivatives such as irinotecan and topotecan (Sriram et al. 2005). In ADCs, camptothecin analogs including SN‐38 (active metabolite of irinotecan) and exatecan (used in trastuzumab deruxtecan) are used as payloads (*NCI Drug Dictionary. fam‐trastuzumab deruxtecan‐nxki*; Ramesh et al. 2010). The cytotoxic effect of camptothecin relies on the inhibition of DNA topo‐isomerase I, which plays a role in DNA replication. This enzyme normally unwinds DNA coils by cleaving one DNA strand, unwinding it and resealing it. Topo‐isomerase I inhibitors bind to the topo‐isomerase I‐DNA complex before resealing the DNA, thereby causing single‐stranded DNA breaks. These are converted into double‐strand DNA breaks when DNA replication is attempted. Accumulation of double‐stranded DNA breaks will ultimately lead to apoptosis of the cell (Sriram et al. 2005; Theocharopoulos et al. 2021).

## 3 Literature Search

### 3.1 Drug Selection

In this review, we focused on available LC–MS methods quantifying different ADC entities for all ADCs approved or under review by the EMA. Compared with approved drugs by the FDA, two additional ADCs could be included by selecting EMA approved drugs. This resulted in 10 ADCs with current market authorization and two ADCs under review by January 2025. Therefore, 12 ADCs were covered in this review: brentuximab vedotin (Adcetris), enfortumab vedotin (Padcev), polatuzumab vedotin (Polivy), tisotumab vedotin (Tivdak), belantamab mafodotin (Blenrep), gemtuzumab ozogamicin (Mylotarg), inotuzumab ozogamicin (Besponsa), sacituzumab govitecan (Trodelvy), trastuzumab deruxtecan (Enhertu), loncastuximab tesirine (Zynlonta), mirvetuximab soravtansine (Elahere), and trastuzumab emtansine (Kadcyla).

### 3.2 Search Terms and Inclusion of Literature

All ADCs have different properties and entities, and therefore literature for each ADC individually was searched. PubMed was searched on January 10, 2025 for each ADC based on their brand name, drug name, and drug development name used in clinical trials. Title and abstracts were screened on these names including different spellings and the presence of “LC–MS.”

This resulted in the following search terms per ADC:

*((“ADC brand name” [Title/Abstract] OR (“drug name” [Title/Abstract]) OR (“drug‐name” [Title/Abstract]) OR (“drug development name”) [Title/Abstract]) AND (“LC‐MS” [Title/Abstract])*.

Then, every article was screened for inclusion or exclusion in this review. Inclusion criteria included registered ADCs and a quantitative LC–MS method.

In addition to the PubMed literature search, the FDA Multidiscipline and Clinical Pharmacology Review per ADC were examined, as they often provide more unpublished bioanalytical method information than the EMA approval documents. When LC–MS methods were described, these were included as well.

## 4 LC–MS Methods Per ADC Entity

**FIGURE 3:**
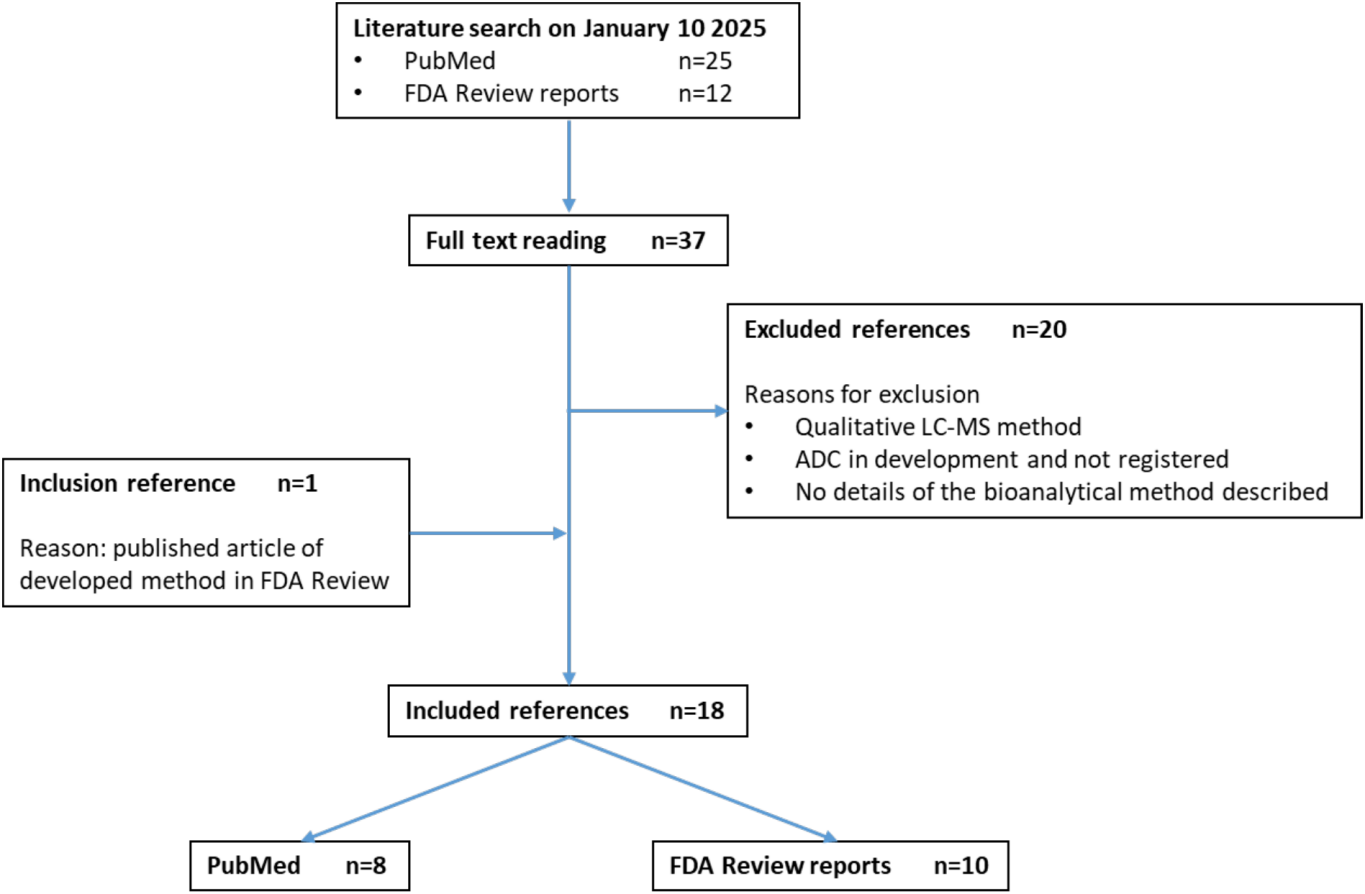
Flowchart of the literature inclusion process for the review. FDA Review refers to both the FDA Multi‐Disciplinary Review and the FDA Clinical Pharmacology Biopharmaceutics Review(s).

A flowchart of the literature search is displayed in Figure 3. After full reading of the articles, 18 references were included in this review.

Results are grouped by the analyzed entities of the ADC, which could be either the intact ADC, the antibody alone (total antibody), (amino acid)‐linker‐payload, and the conjugated, unconjugated or total payload.

Table 1 displays details of the ADCs that were included in the literature search with their product name, target, IgG‐isotype, type of conjugation, linker, payload, DAR, and the chemical structure of the linker‐payload. Results of the literature search are summarized in Tables 2, 3, 4. In Table 2, per analyzed entity of the ADC, the sample pre‐treatment, chromatography and mass spectrometry details are shown. In Tables 3 and 4, the results of assessed validation experiments, including stability results, are presented. Key findings from these studies are described below.

**TABLE 1:** A detailed overview of the ADCs included in the literature search: product name, target, IgG‐isotype, type of conjugation, linker, payload, drug‐to‐antibody ratio, and the chemical structure of the conjugated molecule (linker‐payload).

Antibody‐drug conjugate (product name) (References)	Target	IgG‐isotype	Conjugation	Linker	Payload	Drug‐to‐antibody ratio (DAR)	Chemical structure linker‐payload before conjugation to the antibody. Dashed line separates the linker and payload indicated with curly brackets. Structures were created with Marvin from Chemaxon based on the MOLfiles of the linker and payload and checked in literature.
Trastuzumab emtansine (Kadcyla) (*European Medicines Agency. Summary of Product Characteristics—KADCYLA*; Tong et al. 2021; *U.S. Food & Drug Administration. Drug Approval Package: KADCYLA—Clinical Pharmacology and Biopharmaceutics Review(s)*)	HER2	IgG1	Lysine	Non‐cleavable thio‐ether maleimidomethyl cyclohexane‐1‐carboxylate (MCC) linker	Mertansine (DM1)	3–4	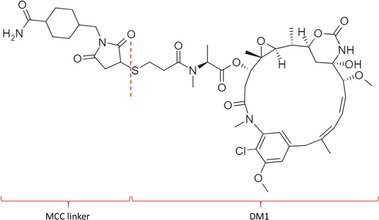
Belantamab mafodotin (Blenrep) (*European Medicines Agency. Assesment Report—BLENREP*; *European Medicines Agency. Summary of Product Characteristics—BLENREP*; Ketchum et al. 2022)	B‐cell maturation antigen (BCMA)	IgG1	Cysteine	Non‐cleavable protease resistant maleimidocapryol (MC) linker	Monomethyl auristatin F (MMAF)	4	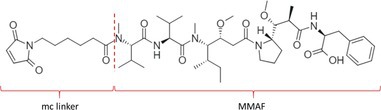
Polatuzumab vedotin (Polivy) (*European Medicines Agency. Assessment report—POLIVY*; *European Medicines Agency. Summary of Product Characteristics—POLIVY*)	CD79b on B‐cells	IgG1	Cysteine	Cleavable protease‐sensitive maleimidocaproyl valine‐citrulline dipeptide linker with a self‐immolative para‐aminobenzylcarbamate (PABC) spacer	Monomethyl auristatin E (MMAE)	3.5	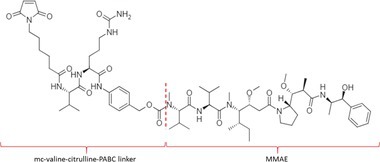
Inotuzumab ozogamicin (Besponsa) (Bouchard et al. 2014; *European Medicines Agency. Summary of Product Characteristics—BESPONSA*; Tong et al. 2021; Yurkiewicz et al. 2018)	CD22 on B‐cells	IgG4	Lysine	Cleavable acid‐labile 4‐(4‐acetylphenoxy)butanoic acid (AcBut) hydrazine linker	*N*‐acetyl‐γ‐calicheamicin	4	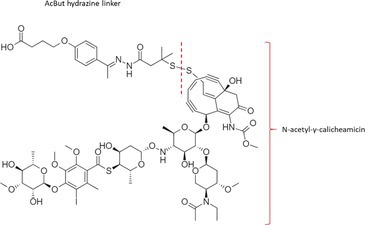
Brentuximab vedotin (Adcetris) (*European Medicines Agency. Summary of Product Characteristics—ADCETRIS*; Tong et al. 2021; van de Donk and Dhimolea 2012)	CD30 on T‐cells	IgG1	Cysteine	Cleavable maleimidocaproyl protease‐sensitive valine‐citrulline dipeptide linker with a self‐immolative PABC spacer	MMAE	4	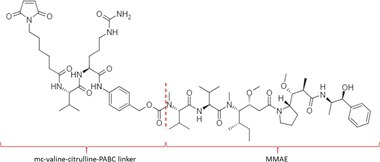
Tisotumab vedotin (Tivdak) (*Tivdak. Mechanism of Action—Tivdak: the first‐and‐only antibody‐drug conjugate (ADC) for adults with previously recurrent or metastatic cervical cancer*; *U.S. Food & Drug Administration. Highlights of prescribing information—TIVDAK*)	Tissue factor (TF)	IgG1	Cysteine	Cleavable maleimidocaproyl protease‐sensitive valine‐citrulline dipeptide linker with a self‐immolative PABC spacer	MMAE	4	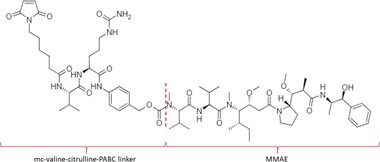
Enfortumab vedotin (Padcev) (*European Medicines Agency. Assessment report—PADCEV*; *European Medicines Agency. Summary of Product Characteristics—PADCEV*)	Nectin‐4	IgG1	Cysteine	Cleavable maleimidocaproyl protease‐sensitive valine‐citrulline dipeptide linker with a self‐immolative PABC spacer	MMAE	4	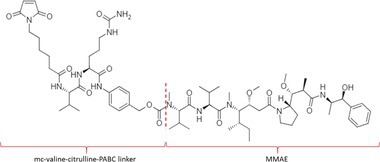
Sacituzumab govitecan (Trodelvy) (*European Medicines Agency. Assessment Report—TRODELVY*; *European Medicines Agency. Summary of Product Characteristics—TRODELVY*; Jain et al. 2015; Kopp et al. 2023; Tong et al. 2021)	Trop‐2	IgG1	Cysteine	Cleavable acid‐sensitive hydrolysable linker (CL2A)	SN‐38	7–8	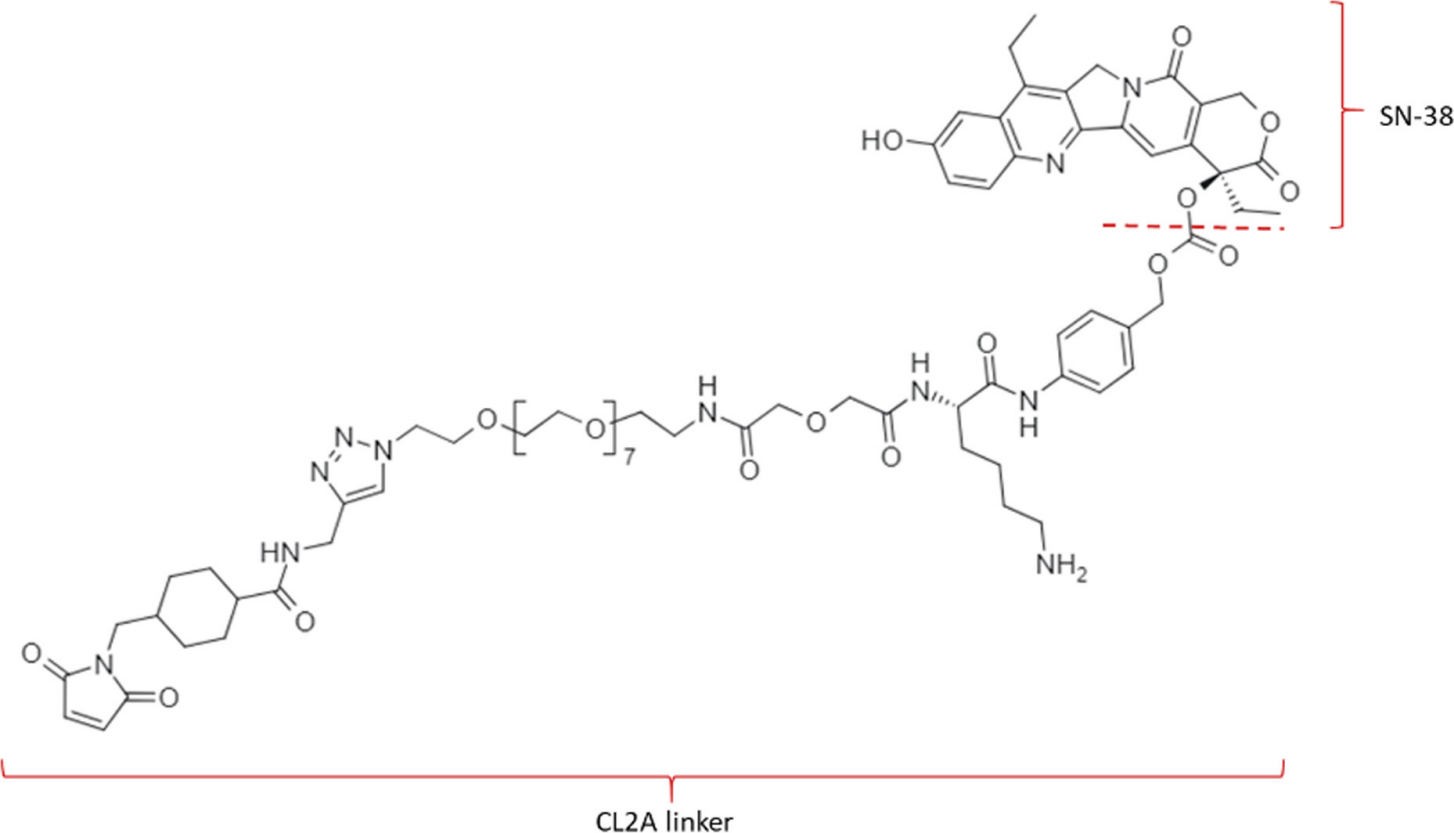
Trastuzumab deruxtecan (Enhertu) (Balamkundu and Liu 2023; *European Medicines Agency. Summary of Product Characteristics—ENHERTU*; *U.S. Food & Drug Administration (FDA). Highlights of prescribing information—ENHERTU*)	HER2	IgG1	Cysteine	Cleavable maleimidocaproyl glycine–glycine‐phenylalanine‐glycine (GGFG) tetrapeptidyl‐aminomethoxy linker	Exatecan derivative DXd	8	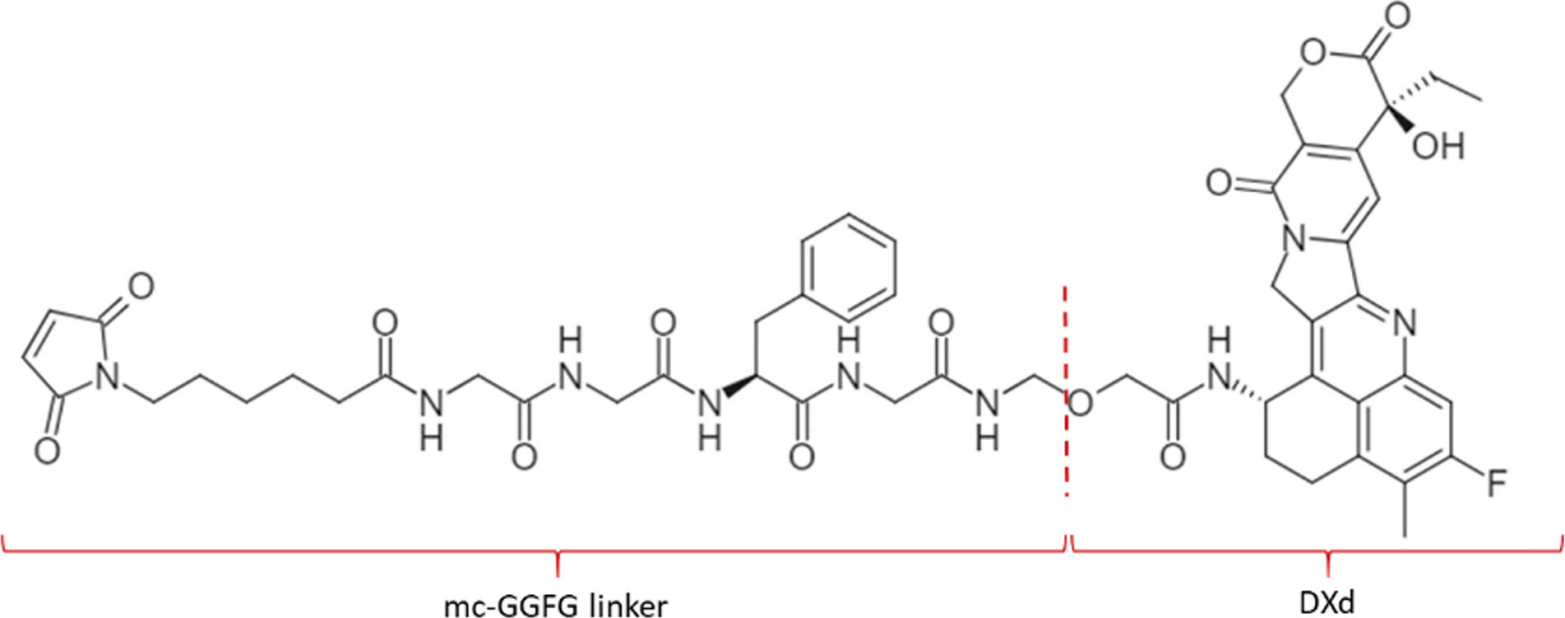
Loncastuximab tesirine (Zynlonta) (*European Medicines Agency. Assessment Report—ZYNLONTA*; *European Medicines Agency. Summary of Product Characteristics—ZYNLONTA*)	CD19 on B‐cells	IgG1	Cysteine	Cleavable SG3249 PBD drug linker consisting of a maleimide‐poly (ethylene glycol) (PEG)8 spacer‐protease‐sensitive valine‐alanine dipeptide linker with a self‐immolative PABC spacer	SG‐3199	2.3	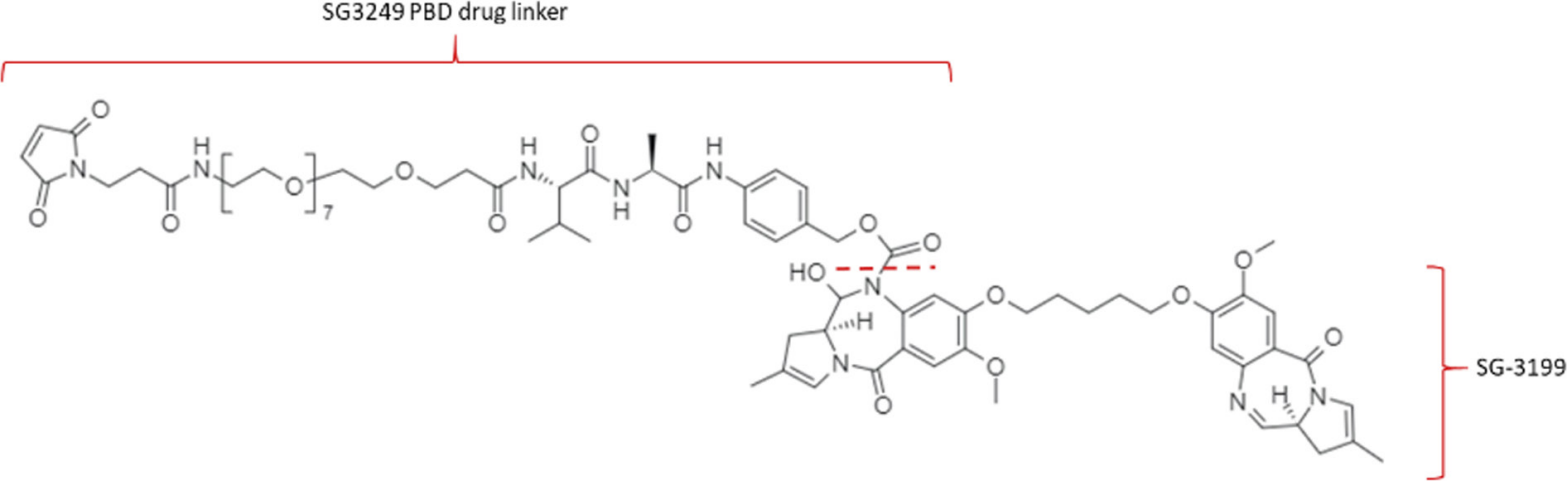
Mirvetuximab soravtansine (Elahere) (Heo 2023; Nwabufo 2023; *U.S. Food & Drug Administration. Highlights of prescribing information—ELAHERE*)	Folate receptor‐α	IgG1	Lysine	Cleavable sulpho‐SPDB linker	Maytansinoid (DM4)	3.4	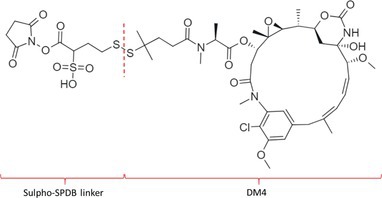
Gemtuzumab ozogamicin (Mylotarg) (*European Medicines Agency. Summary of Product Characteristics—MYLOTARG*; Fu et al. 2022; Tong et al. 2021)	CD33	IgG4	Lysine	Cleavable acid‐labile AcBut [4‐(4‐acetylphenoxy)butanoic acid] hydrazine linker	*N*‐acetyl‐γ‐calicheamicin	2–3	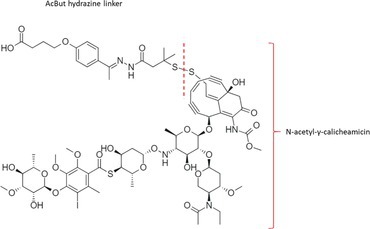

**TABLE 2:** Sample pre‐treatment, chromatography, and mass spectrometry details of the LC–MS methods of the ADC entity.

Drug name	Analyte	Internal standard (IS)	Sampling volume	Matrix	Sample pre‐treatment	Chromatography		Mass spectrometry		References
						LC column	Mobile phase	Detector	Mass transitions	
Intact ADC										
Trastuzumab emtansine	Trastuzumab emtansine	—	100 μL	Rat plasma	Immuno‐affinity purification	Waters Acquity Protein BEH C4, 300 Å, 1.7 μm, 2.1 × 50 mm column	A: 0.1% formic acid in water B: 0.1% formic acid in acetonitrile	SCIEX X500B QTOF mass spectrometer coupled with an ExionLC AD	—	(Jin et al. 2018)
Total antibody										
Trastuzumab emtansine	Signature peptide IYPTNGYTR (as surrogate for total antibody trastuzumab)	P_14_R synthetic peptide	10 μL	Plasma	Nano‐surface and molecular‐orientation limited (nSMOL)	Shim‐pack GISS C18 (2.1 × 50 mm, 1.9 μm, SHIMADZU)	A: 0.1% aqueous formic acid B: acetonitrile with 0.1% formic acid	Triple quadrupole LCMS‐8050 (SHIMADZU) with Nexera X2 gradient pumps	IYPTNGYTR: m/z 542.8 ➔ 404.7 [M + 2H]^2+^ P_14_R: m/z 512.1 ➔ 292.3 [M + H]^+^	(Iwamoto et al. 2017)
Amino acid‐linker‐payload										
Trastuzumab emtansine	Intracellular lysine‐MCC‐DM1	Ansamitocin P‐3	200 μL	Drug‐free cell suspension	Protein precipitation ➔ nitrogen evaporation ➔ reconstitution	Phenomenex Kinetex C18 column (100 × 2.1 mm, 2.6 μm)	A: 0.1% (v/v) formic acid in deionized water B: 0.1% (v/v) formic acid in acetonitrile	SCIEX API 4000 triple‐quadrupole mass spectrometer	Lys‐MCC‐DM1: m/z 1103.6 ➔ 485.3 Ansamitocin P‐3: m/z 635.3 ➔ 547.3	(Liu et al. 2017)
Belantamab mafodotin	Cyclic form of cysteine‐mcMMAF	[^15^N, ^13^C_3_, ^2^H_3_] cysteine‐mc‐MMAF	100 μL	Plasma	Solid phase extraction	Waters BEH C18 (1.7 μm, 100 × 2.1 mm i.d.)	—	Waters ACQUITY UPLC and Xevo TQ‐S tandem TQ‐MS with ESI	m/z 523.8 ➔ 619.4 [M + 2H]^2+^	(Mayer et al. 2021)
Belantamab mafodotin	Cyclic form of cysteine‐mcMMAF	[^15^N, ^13^C_3_, ^2^H_3_] cysteine‐mc‐MMAF	50 μL	Plasma	Acidic protein precipitation	Waters HSS T3 (1.8 μm, 100 × 2.1 mm i.d.)	—	Waters ACQUITY UPLC and Xevo TQ‐S tandem TQ‐MS with ESI	m/z 523.8 ➔ 619.4 [M + 2H]^2+^	(Mayer et al. 2021)
Linker‐payload										
Trastuzumab emtansine	MCC‐DM1	Ansamitocin P‐3	40 μL	Plasma	Liquid–liquid extraction	Shim‐pack GISS C18 (2.1 × 50 mm, 1.9 μm, SHIMADZU)	A: distilled water B: methanol	Triple quadrupole LCMS‐8050 (SHIMADZU) with Nexera X2 gradient pumps	DM1‐MCC: m/z 975.1 ➔ 547.2 Ansamitocin P‐3: m/z 635.2 ➔ 547.2	(Iwamoto et al. 2017)
Trastuzumab emtansine	Intracellular MCC‐DM1	Ansamitocin P‐3	200 μL	Drug‐free cell suspension	Protein precipitation ➔ nitrogen evaporation ➔ reconstitution	Phenomenex Kinetex C18 column (100 × 2.1 mm, 2.6 μm)	A: 0.1% (v/v) formic acid in deionized water B: 0.1% (v/v) formic acid in acetonitrile	SCIEX API 4000 triple‐quadrupole mass spectrometer	MCC‐DM1: m/z 957.7 ➔ 485.3 Ansamitocin P‐3: m/z 635.3 ➔ 547.3	(Liu et al. 2017)
Trastuzumab emtansine	MCC‐DM1	DM1‐NEM Post‐column infusion (PCI)‐IS: maytansine, Lys‐MCC‐DM1, exatecan	200 μL	Plasma	Reduction with dithiotreitol ➔ protein precipitation ➔ free thiol blockage with NEM ➔ nitrogen evaporation ➔ reconstitution ➔ filtered through 0.22 mm cellulose membrane	Agilent ZORBAX Eclipse Plus C18 2.1 × 100 mm (1.8 μm) column	A: 0.1% formic acid and 10 mM ammonium acetate B: 90:10 (v/v) isopropyl alcohol: acetonitrile with 0.1% formic acid and 10 mM ammonium acetate	Agilent 1290 UHPLC coupled to a 6495C triple quadrupole system	MCC‐DM1: ‐quantifier: m/z 957.4 ➔ 485.2 ‐qualifier: m/z 957.4 ➔ 913.4 DM1‐NEM: ‐quantifier: m/z 845.3 ➔ 485.2 ‐qualifier: m/z 845.3 ➔ 801.3 Maytansine PCI‐IS: m/z 674.3 ➔ 485.2 Lys‐MCC‐DM1 PCI‐IS: m/z 1103.5 ➔ 10.41.5 Exatecan PCI‐IS: m/z 436.2 ➔ 375.1	(Cheng et al. 2024)
Payload										
*Conjugated*										
Polatuzumab vedotin	Conjugated MMAE	ADC‐IS with released MMAF‐IS	—	Plasma	Immuno‐affinity capture ➔ papain digestion ➔ nitrogen evaporation ➔ protein precipitation ➔ nitrogen evaporation ➔ reconstitution	—	—	LC–MS/MS with positive ion electrospray	MMAE: m/z 718.7 ➔ 152.2 MMAF‐IS: m/z 732.7 ➔ 170.2	(*U.S. Food & Drug Administration. Drug Approval Package: POLIVY—Multi‐Discipline Review*)
Inotuzumab ozogamicin	Conjugated calicheamicin (as *N*‐acetyl‐ε‐calicheamicin)	*N*‐propionyl‐γ‐calicheamicin dimethyl hydrazide (DMH) (as *N*‐propionyl‐ε‐calicheamicin)	—	Plasma	Methyl tert‐butyl ether (MTBE) extraction ➔ reduction with dithiotreitol ➔ MTBE extraction	—	—	RP‐HPLC–MS/MS with positive ion electrospray	—	(*U.S. Food & Drug Administration. Drug Approval Package: BESPONSA—Multi‐Discipline Review*)
Trastuzumab emtansine	Conjugated DM1	—	—	Mouse plasma	Immuno‐affinity capture ➔ acid elution ➔ reduction with dithiotreitol and deglycosylation	Thermo Fisher Scientific MabPac column (PN088648)	A: 0.1% formic acid in water B: 0.1% formic acid in acetonitrile	SCIEX Exion LC coupled to ZenoTOF7600	Precursor ions: ‐Light chain with 1 (LC1) payload: 1356^18+^, 1436^17+^ ‐Light chain with 2 payloads (LC2): 1409.6^18+^ Product ion for quantification: 547.2^+^	(Yuan et al. 2024)
*Unconjugated*										
Brentuximab vedotin	Unconjugated MMAE (free and non‐specifically bound)	MMAE‐D_8_	—	Plasma Urine Feces	Solid phase extraction	—	—	—	—	(*U.S. Food & Drug Administration. Drug Approval Package: ADCETRIS—Multi‐Discipline Review*)
Tisotumab vedotin	Unconjugated MMAE	MMAE‐D_8_	—	Plasma	Solid phase extraction	—	—	—	—	(*U.S. Food & Drug Administration. Drug Approval Package: TIVDAK—Multi‐Discipline Review*)
Enfortumab vedotin	Free MMAE	—	25 μL	Plasma (sodium citrate)	Supported liquid extraction	—	—	—	—	(*U.S. Food & Drug Administration. Drug Approval Package: PADCEV—Multi‐Discipline Review*)
Polatuzumab vedotin	Unconjugated MMAE	MMAE‐D_8_	—	Plasma (lithium heparin)	Protein precipitation ➔ nitrogen evaporation ➔ reconstitution	—	—	LC–MS/MS with positive ion electrospray	MMAE: m/z 718.6 ➔ 152.2 MMAE‐D_8_: m/z 726.6 ➔ 152.2	(*U.S. Food & Drug Administration. Drug Approval Package: POLIVY—Multi‐Discipline Review*)
Inotuzumab ozogamicin	Unconjugated calicheamicin (as *N*‐acetyl‐γ‐calicheamicin DMH)	*N*‐propionyl‐γ‐calicheamicin DMH	—	Serum	Solid phase extraction	—	—	RP‐HPLC–MS/MS with positive ion electrospray	—	(*U.S. Food & Drug Administration. Drug Approval Package: BESPONSA—Multi‐Discipline Review*)
Sacituzumab govitecan	Free SN‐38 and SN‐38G	SN‐38‐D_3_ SN‐38G‐^13^C_6_	50 μL	Serum	Solid phase extraction under yellow light	—	—	—	—	(*U.S. Food & Drug Administration. Drug Approval Package: TRODELVY—Multi‐Discipline Review*)
Trastuzumab deruxtecan	Free DXd	MAAA‐1454	—	Serum	Deproteinization by organic solvents and filtration ➔ evaporation ➔ reconstitution	—	—	RP‐HPLC‐MS/MS with positive ion electrospray	—	(*U.S. Food & Drug Administration. Drug Approval Package: ENHERTU—Multi‐Discipline Review*)
Trastuzumab deruxtecan	Free DXd	IS‐1: SN‐38 IS‐2: SN‐38G Post‐column infusion (PCI)‐IS: maytansine, Lys‐MCC‐DM1, exatecan	200 μL	Plasma	Reduction with dithiotreitol ➔ protein precipitation ➔ free thiol blockage with NEM ➔ nitrogen evaporation ➔ reconstitution ➔ filtered through 0.22 mm cellulose membrane	Agilent ZORBAX Eclipse Plus C18 2.1 × 100 mm (1.8 μm) column	A: 0.1% formic acid and 10 mM ammonium acetate B: 90:10 (v/v) isopropyl alcohol: acetonitrile with 0.1% formic acid and 10 mM ammonium acetate	Agilent 1290 UHPLC coupled to a 6495C triple quadrupole system	DXd: ‐quantifier: m/z 494.2 ➔ 419.1 ‐qualifier: m/z 494.2 ➔ 375.1 SN‐38: ‐quantifier: m/z 393.1 ➔ 234.0 ‐qualifier: m/z 393.1 ➔ 249.0 SN‐38G: ‐quantifier: m/z 569.2 ➔ 3489.1 ‐qualifier: m/z 569.2 ➔ 393.1 Maytansine PCI‐IS: m/z 674.3 ➔ 485.2 Lys‐MCC‐DM1 PCI‐IS: m/z 1103.5 ➔ 10.41.5 Exatecan PCI‐IS: m/z 436.2 ➔ 375.1	(Cheng et al. 2024)
Loncastuximab tesirine	Free SG3199	SG3199‐D_10_	100 μL	Serum	Solid phase extraction	—	—	—	—	(*U.S. Food & Drug Administration. Drug Approval Package: ZYNLONTA—Multi‐Discipline Review*)
Mirvetuximab soravtansine	DM4 and S‐methyl DM4 (DM4‐Me)	—	—	Plasma (K_2_EDTA with citric acid)	—	—	—	—	—	(*U.S. Food & Drug Administration. Drug Approval Package: ELAHERE—Multi‐Discipline Review*)
Trastuzumab emtansine	Free and disulfide‐bound mertansine (DM1) (measured as *N*‐ethyl maleimide (DM1‐NEM))	Maytansine	30 μL	Plasma	Reduction with Tris(2‐carboxyethyl) phosphine ➔ protein precipitation ➔ free thiol blockage with NEM ➔ online solid phase extraction	Synergi MAX‐RP 80A (50 × 1.0 mm, 4 μm)	A: 5 mM ammonium acetate with 0.1% formic acid in H_2_O B: 5 mM ammonium acetate with 0.1% formic acid in 95:5 acetonitrile:H_2_O	API3000 (AB Sciex, MA, USA) in SRM positive ionization mode	—	(Dere et al. 2013)
Trastuzumab emtansine	Free DM1 (as DM1‐NEM)	Maytansine	30 μL	Rat plasma	Reduction with Tris(2‐carboxyethyl) phosphine ➔ free thiol blockage with NEM	Atlantis dC18 column (3 μm, 2.1 mm × 50 mm, Waters Corp.)	A: 5 mM ammonium acetate with 0.1% formic acid in H_2_O B: 5 mM ammonium acetate with 0.1% formic acid in 95:5 acetonitrile:H_2_O	API 5000 mass spectrometer coupled with an Agilent 1260 HPLC system.	DM1‐NEM: m/z 845.4 ➔ 485.3. Maytansine: m/z 692.2 ➔ 547.2	(Jeon et al. 2023)
Trastuzumab emtansine	DM1	Ansamitocin P‐3	200 μL	Drug‐free cell suspension	Protein precipitation ➔ nitrogen evaporation ➔ reconstitution	Phenomenex Kinetex C18 column (100 × 2.1 mm, 2.6 μm)	A: 0.1% (v/v) formic acid in deionized water B: 0.1% (v/v) formic acid in acetonitrile	SCIEX API 4000 triple‐quadrupole mass spectrometer	DM1: m/z 738.5 ➔ 547.5 Ansamitocin P‐3: 635.3 ➔ 547.3	(Liu et al. 2017)
Trastuzumab emtansine	Free DM1 (as DM1‐NEM)	MCC‐DM1 Post‐column infusion (PCI)‐IS: maytansine, Lys‐MCC‐DM1, exatecan	200 μL	Plasma	Reduction with dithiotreitol ➔ protein precipitation ➔ free thiol blockage with NEM ➔ nitrogen evaporation ➔ reconstitution ➔ filtered through 0.22 mm cellulose membrane	Agilent ZORBAX Eclipse Plus C18 2.1 × 100 mm (1.8 μm) column	A: 0.1% formic acid and 10 mM ammonium acetate B: 90:10 (v/v) isopropyl alcohol: acetonitrile with 0.1% formic acid and 10 mM ammonium acetate	Agilent 1290 UHPLC coupled to a 6495C triple quadrupole system	DM1‐NEM: ‐quantifier: m/z 845.3 ➔ 485.2 ‐qualifier: m/z 845.3 ➔ 801.3 MCC‐DM1: ‐quantifier: m/z 957.4 ➔ 485.2 ‐qualifier: m/z 957.4 ➔ 913.4 Maytansine PCI‐IS: m/z 674.3 ➔ 485.2 Lys‐MCC‐DM1 PCI‐IS: m/z 1103.5 ➔ 10.41.5 Exatecan PCI‐IS: m/z 436.2 ➔ 375.1	(Cheng et al. 2024)
Sacituzumab govitecan	SN‐38 and SN‐38 G	SN‐38: ‐IS‐1 DXd ‐IS‐2 SN‐38G SN‐38G ‐IS‐1 Dxd ‐IS‐2 SN‐38 Post‐column infusion (PCI)‐IS: maytansine, Lys‐MCC‐DM1, exatecan	200 μL	Plasma	Reduction with dithiotreitol ➔ protein precipitation ➔ free thiol blockage with NEM ➔ nitrogen evaporation ➔ reconstitution ➔ filtered through 0.22 mm cellulose membrane	Agilent ZORBAX Eclipse Plus C18 2.1 × 100 mm (1.8 μm) column	A: 0.1% formic acid and 10 mM ammonium acetate B: 90:10 (v/v) isopropyl alcohol: acetonitrile with 0.1% formic acid and 10 mM ammonium acetate	Agilent 1290 UHPLC coupled to a 6495C triple quadrupole system	SN‐38: ‐quantifier: m/z 393.1 ➔ 234.0 ‐qualifier: m/z 393.1 ➔ 249.0 SN‐38G: ‐quantifier: m/z 569.2 ➔ 3489.1 ‐qualifier: m/z 569.2 ➔ 393.1 DXd: ‐quantifier: m/z 494.2 ➔ 419.1 ‐qualifier: m/z 494.2 ➔ 375.1 Maytansine PCI‐IS: m/z 674.3 ➔ 485.2 Lys‐MCC‐DM1 PCI‐IS: m/z 1103.5 ➔ 10.41.5 Exatecan PCI‐IS: m/z 436.2 ➔ 375.1	(Cheng et al. 2024)
Total payload										
Sacituzumab govitecan	Total SN‐38 (free and acid‐dissociated SN‐38)	SN‐38‐D_3_	50 μL	Serum	Hydrolysis ➔ protein precipitation	—	—	—	—	(*U.S. Food & Drug Administration. Drug Approval Package: TRODELVY—Multi‐Discipline Review*)

*Note:* — means that no data on this topic was available or reported.

Abbreviation: NEM means *N*‐ethylmaleimide.

**TABLE 3:** Validation results for each LC–MS method per ADC entity, including analyte, calibration data, accuracy and precision, sensitivity, selectivity, carry‐over, dilution integrity, matrix effect or factor, and recovery results.

Drug name	Analyte	Calibration		Accuracy and precision		LLOQ	Selectivity		Carry‐over	Dilution integrity	Matrix effect or factor	Recovery	References
		Levels	Range	Levels	Results	S/N	Endogenous	Exogenous					
Intact ADC													
Trastuzumab emtansine	53+ charge state of trastuzumab emtansine DAR 3 G0F‐1/G1F‐1	5	5–100 μg/mL	—	—	—	—	—	—	—	—	70%	(Jin et al. 2018)
Trastuzumab emtansine	DAR 3; G0F‐2	6	5–100 μg/mL	5, 10, 20, 50, 100 μg/mL	Accuracy range: 89.3%–110.2%	—	—	—	—	—	—	—	(Jin et al. 2018)
Trastuzumab emtansine	DAR 3; G0F‐1/G1F‐1	6	5–100 μg/mL	5, 10, 20, 50, 100 μg/mL	Accuracy range: 89.3%–110.2%	—	—	—	—	—	—	—	(Jin et al. 2018)
Trastuzumab emtansine	DAR 3; G0F‐1/G2F‐1 or G1F‐2	6	5–100 μg/mL	5, 10, 20, 50, 100 μg/mL	Accuracy range: 91.5%–109.1%	—	—	—	—	—	—	—	(Jin et al. 2018)
Trastuzumab emtansine	DAR 3	6	5–100 μg/mL	5, 10, 20, 50, 100 μg/mL	Accuracy range: 89.5%–109.6%	—	—	—	—	—	—	—	(Jin et al. 2018)
Trastuzumab emtansine	All DARs	6	5–100 μg/mL	5, 10, 20, 50, 100 μg/mL	Accuracy range: 90.5%–108.3%	—	—	—	—	—	—	—	(Jin et al. 2018)
Total antibody													
Trastuzumab emtansine	Signature peptide IYPTNGYTR (as surrogate for total antibody trastuzumab)	6	0.061–250 μg/mL T‐DM1	0.061, 2.93, 23.4, and 200 μg/mL	Mean accuracy: 91%–115% Overall precision: 2.23%–8.35%	—	—	—	—	—	—	—	(Iwamoto et al. 2017)
Amino acid‐linker‐payload													
Trastuzumab emtansine	Lys‐MCC‐DM1	—	1.00–500 nM	All concentrations	Overall precision: ≤ 6.8% Intra‐run precision: ≤ 8.6%	—	—	—	—	—	—	—	*(U.S. Food & Drug Administration. Drug Approval Package: KADCYLA ‐ Clinical Pharmacology and Biopharmaceutics Review(s))*
Trastuzumab emtansine	Intracellular Lys‐MCC‐DM1	7	1–100 nM Lys‐MCC	2, 20, and 100 nM	Accuracy (RE, %): 6.4–9.4 Intra‐run precision: 3.5%–6.9% Inter‐run precision: 7.9%–8.1%	—	No significant interference in blank lots	No significant interference	—	—	96.8%–100.5% (CV% 4.3‐6.9)	87.8%–92.6% (CV% 4.5‐12.6)	(Liu et al. 2017)
Belantamab mafodotin	Cyclic form of cysteine‐mcMMAF	—	50–10,000 pg/mL	500 pg/mL Cys‐mcMMAF and 150 μg/mL BMF	Mean concentration: 766.0 pg/mL (bias 53.2%, CV 7.2%)	—	—	—	—	—	—	—	(Mayer et al. 2021)
Belantamab mafodotin	Cyclic form of cysteine‐mcMMAF	—	50‐10,000 pg/mL	150 pg/mL Cys‐mcMMAF and 150 μg/mL BMF	Mean concentration: 158.5 pg/mL (bias 5.9%, CV 2.7%)	—	—	—	—	—	—	—	(Mayer et al. 2021)
				500 pg/mL Cys‐mcMMAF and 150 μg/mL BMF	Mean concentration: 510.1 pg/mL (bias 2.0%, CV 1.7%)								
				1000 pg/mL Cys‐mcMMAF and 150 μg/mL BMF	Mean concentration: 1,000.1 pg/mL (bias 0.01%, CV 2.1%)								
				All three concentration levels	Accuracy: ≥ 5.9% Precision: ≥ 2.7%								
Linker‐payload													
Trastuzumab emtansine	MCC‐DM1	10	0.391‐200 ng/mL MCC‐DM1	0.391, 1.17, 18.8, and 150 ng/mL	Mean accuracy: 91.3%–102% Overall precision: 2.56%–4.98%	—	—	—	No carry over detected	50‐fold of 500 ng/mL	IS‐normalized matrix factor at 1.17 ng/mL: 14.9 (CV% 7.01) IS‐normalized matrix factor at 150 ng/mL: 6.74 (CV% 3.20)	Repeat recovery rate P‐3: 99.1%	(Iwamoto et al. 2017)
Trastuzumab emtansine	Intracellular MCC‐DM1	7	1‐100 nM MCC‐DM1	2, 20, and 100 nM	Accuracy (RE,%): −6.6 to −0.2 Intra‐run precision: 2.6%–6.8% Inter‐run precision: 4.7%–5.6%	—	No significant interference in blank lots	No significant interference	—	—	96.6%–107.6% (CV% 5.2‐5.9)	91.4%–110.0% (CV% 3.9‐5.2)	(Liu et al. 2017)
Trastuzumab emtansine	MCC‐DM1	6	0.5–20 ng/mL	0.5, 1.5, 7.5, 15 ng/mL	Results based on PCI‐IS Intra‐run accuracy: 91.9% (±2.0%)–111.3% (±2.9%) Intra‐run precision (RSD%): < 5.3% Inter‐run accuracy: 90.2% (±2.8%)–113.7% (±7.5%) Inter‐run precision (RSD%): < 10.9%	—	—	—	—	—	CV% ‐No IS correction: 13% ‐PCI‐IS: 4% ‐Normal IS: 7%	—	(Cheng et al. 2024)
Payload													
*Conjugated*													
Polatuzumab vedotin	Conjugated MMAE	—	0.5–50.0 nM	LLOQ (0.5 nM)	Intra‐run accuracy: −6.23 to 6.19% Intra‐run precision: < 10.4% Inter‐run accuracy: −2.24% Inter‐run precision: 15.6%	—	No significant interference in blanks	—	—	Twofold for 5.00 nM and 1000‐fold for 8000 nM polatu‐zumab vedotin	—	Analyte: 104%–116% across QCL, QCM, QCH IS: 45%–49%	*(U.S. Food & Drug Administration. Drug Approval Package: POLIVY ‐ Multi‐Discipline Review)*
				Other QC levels (1.00, 5.00, 12.0, and 39.0 nM)	Intra‐run accuracy: −2.3% to 5.04% Intra‐run precision: < 5.66% Inter‐run accuracy: −2.1% to 0.1% Inter‐run precision: 3.4%–8.82%								
Inotuzumab ozogamicin	*N*‐acetyl‐ε‐calicheamicin as surrogate for inotuzumab ozogamicin	—	1.00‐500 ng/mL	LLOQ QC (1.00 ng/mL)	Intra‐run accuracy: −13.7%–6.19% Intra‐run precision: < 15.3% Inter‐run accuracy: 0.821% Inter‐run precision: 16.3%	—	No response in 10 blank lots	—	—	Tenfold	—	Analyte: 49.2% across QCL, QCM, QCH IS: 126%	*(U.S. Food & Drug Administration. Drug Approval Package: BESPONSA ‐ Multi‐Discipline Review)*
				All other QC levels (2.50, 25.0, 200, 375, and 1,000 ng/mL)	Intra‐run accuracy: −6.8% to 17.2% Intra‐run precision: < 16.3% Inter‐run accuracy: 1.65%–10.2% Inter‐run precision: 8.29%–10.5%								
Trastuzumab emtansine	Conjugated DM1	LC1: 9 LC2: 7	LC1: 3000–90,000 ng/mL LC2: 9000‐90,000 ng/mL	—	Acceptance criteria ±20% at non‐LLOQ levels and ±25% at LLOQ LC1: 83.3% of 24 QC replicates passed with a CV% of 7.46%. LC2: 77.8% of 18 QC replicates passed with a CV% of 6.82%	> 10	—	—	—	—	—	—	(Yuan et al. 2024)
*Unconjugated*													
Brentuximab vedotin	MMAE	—	*Plasma* 0.025–1.00 ng/mL	CALs	Accuracy: 95.4%–103.6%	—	—	*Plasma*: mean RSD of 2.5% and mean accuracy of 96.0%	—	—	—	—	*(U.S. Food & Drug Administration. Drug Approval Package: ADCETRIS ‐ Multi‐Discipline Review)*
				QC samples	Accuracy: 92.0%–94.7% Inter‐ and intra‐assay precision: ≤ 6%.								
			*Urine* 0.1–50 ng/mL	QC samples (0.300, 5.00, and 40.0 ng/mL)	Accuracy: 101.8%–108% Inter‐ and intra‐assay precision: ≤ 3.8%.			*Urine*: mean RSD of 2.2% and mean accuracy of 100.7%					
			*Faeces* 5.00–2500 ng/g	CALs	Accuracy CALs: 95.4%–103.6%			*Faeces*: mean RSD of 9.3% and mean accuracy of 94.0%					
				QC samples (15.0, 250, 2000 ng/g)	Accuracy: 98.5%–105.3% Inter‐ and intra‐assay precision: ≤ 4.6%								
Tisotumab vedotin	Unconjugated MMAE	8	25–10,000 pg/mL	LLOQ	Intra‐run accuracy: −8.0% Intra‐run precision: 10.0%	—	6 lots blank matrix (CV 2.5%)	—	—	—	Free MMAE: −8.7% MMAE‐d10: −9.9%	—	*(U.S. Food & Drug Administration. Drug Approval Package: TIVDAK ‐ Multi‐Discipline Review)*
				All other (QCL, QCM, QCH	Intra‐run accuracy: −4.2% to 2.7% Intra‐run precision: < 3.3%								
Tisotumab vedotin	Unconjugated MMAE	8	25–10,000 pg/mL	LLOQ	Intra‐run accuracy: −4.9 to −2.7% Intra‐run precision: < 5.0% Inter‐run accuracy: −3.7% Inter‐run precision: 0.0%	—	6 lots of blank matrix (bias ‐9.0%)	—	No observed carry‐over	8000 pg/mL with 10 fold dilution (bias ‐6.1%)	—	—	*(U.S. Food & Drug Administration. Drug Approval Package: TIVDAK ‐ Multi‐Discipline Review)*
				All other (QCL, QCM, QCH	Intra‐run accuracy: −5.7% to 1.0% Intra‐run precision: < 5.5% Inter‐run accuracy: −2.1 to −0.3% Inter‐run precision: < 2.8%								
Enfortumab vedotin	Free MMAE	—	10.0–2,000 pg/mL	—	Intra‐run accuracy: −2.3% to 3.5% Intra‐run precision: 1.0%–7.2% Inter‐run accuracy: −0.1% to 2.0% Inter‐run precision: 2.6%–6.1%	—	—	—	—	2.5‐ and fourfold	—	—	*(U.S. Food & Drug Administration. Drug Approval Package: PADCEV ‐ Multi‐Discipline Review)*
Polatuzumab vedotin	Unconjugated MMAE	8	0.0359–17.9 ng/mL	LLOQ (0.0359 ng/mL), QCL (0.108 ng/mL), QCM (7.18 ng/mL), and QCH (14.4 ng/mL)	Intra‐run accuracy: −0.8% to 10.9% Intra‐run precision: < 8.3% Inter‐run accuracy: < 3.3% Inter‐run precision: < 5.4%	—	—	No significant interference for analyte and IS	—	Tenfold	—	Analyte: 84.1%–87% across QCL, QCM, QCH IS: 67.7%	*(U.S. Food & Drug Administration. Drug Approval Package: POLIVY ‐ Multi‐Discipline Review)*
Inotuzumab ozogamicin	*N*‐acetyl‐γ‐calicheamicin	—	0.05‐10.0 ng/mL	LLOQ QC (0.05 ng/mL)	Intra‐run accuracy: −9.83% to 4.87% Intra‐run precision: < 12.1% Inter‐run accuracy: 2.68% Inter‐run precision: 10.0%	—	No significant interference in 10 blank lots	—	—	Fivefold at 20.0 ng/mL	—	Analyte: 48.6% across QCL, QCM, QCH IS: 67.7%	*(U.S. Food & Drug Administration. Drug Approval Package: BESPONSA ‐ Multi‐Discipline Review)*
				All other QC levels (0.15 and 7.50 ng/mL)	Intra‐run accuracy: −10.3% to 5.62% Intra‐run precision: < 6.71% Inter‐run accuracy: −8.02%–2.68% Inter‐run precision: 4.81%–8.26%								
Sacituzumab govitecan	Free SN‐38	—	1.00‐500 ng/mL	LLOQ	Intra‐run accuracy: 5.6%–8.0% Intra‐run precision: 4.3%–7.7% Inter‐run accuracy: 6.6% Inter‐run precision: 5.6%	—	6 lots interference free	—	≤ 20.0% LLOQ, ≤ 5.0% mean IS	Two‐ and fivefold	IS‐normalized matrix factor: CV% ≤ 15	—	*(U.S. Food & Drug Administration. Drug Approval Package: TRODELVY ‐ Multi‐Discipline Review)*
				Above LLOQ	Intra‐run accuracy: −2.3% to 7.1% Intra‐run precision: 1.2%–5.2% Inter‐run accuracy: −1.1% to 4.8% Inter‐run precision: 1.7%–4.4%								
Sacituzumab govitecan	SN‐38G	—	1.00‐500 ng/mL	LLOQ	Intra‐run accuracy: 6.7%–10.4% Intra‐run precision: 4.3%–8.2% Inter‐run accuracy: 9.0% Inter‐run precision: 5.9%	—	6 lots interference free	—	≤ 20.0% LLOQ, ≤ 5.0% mean IS	Two‐ and fivefold	IS‐normalized matrix factor: CV% ≤ 15	—	*(U.S. Food & Drug Administration. Drug Approval Package: TRODELVY ‐ Multi‐Discipline Review)*
				Above LLOQ	Intra‐run accuracy: 1.0%–4.8% Intra‐run precision: 1.6%–3.2% Inter‐run accuracy: 1.2%–3.7% Inter‐run precision: 2.0%–2.9%								
Trastuzumab deruxtecan	Free DXd	8	10.0‐2000 pg/mL	6 QCs	Cumulative accuracy: −5.09 to −0.244% Inter‐assay precision: ≤ 14.8%	—	No significant interference in 6 blank lots	No significant interference of metabolites on quantitation of MAAA‐1181a	—	Assessed at 4000 and 150 pg/mL but no results are shown	IS‐normalized matrix factor: CV% ≤ 4.18	—	*(U.S. Food & Drug Administration. Drug Approval Package: ENHERTU ‐ Multi‐Discipline Review)*
Trastuzumab deruxtecan	Free DXd	6	0.5–20 ng/mL	0.5, 1.5, 7.5, and 15.0 ng/mL	Results based on PCI‐IS Intra‐run accuracy: 88.2% (±3.0%)–101.6% (±4.0%) Intra‐run precision (RSD%): < 8.0% Inter‐run accuracy: 90.3% (±3.6%)–107.0% (±9.4%) Inter‐run precision (RSD%): < 9.1%	13.0 ± 1.4	—	—	—	—	CV%: ‐No correction: 12% ‐PCI‐IS: 3% ‐Normal IS‐1: 16% ‐Normal IS‐2: 2.5%	—	(Cheng et al. 2024)
Loncastuximab tesirine	Free SG3199	9	25–25,000 pg/mL	25 pg/mL (LLOQ)	Intra‐run accuracy: < 8.5% Intra‐run precision: < 4.8% Inter‐run accuracy: 5.2% Inter‐run precision: 4.9%	—	—	No significant interference for SG3199 and SG3199‐D_10_	Considerable	Tenfold dilution up to 200,000 pg/mL	IS‐normalized matrix factor: 0.953 (CV% 4) and 1.03 (CV% 2.1) for 75 and 20,000 pg/mL, respectively	69.0%–76.7%	*(U.S. Food & Drug Administration. Drug Approval Package: ZYNLONTA ‐ Multi‐Discipline Review)*
				75 (QCL), 1250 (QCM), 20,000 (QCH) pg/mL	Intra‐run accuracy: −4.6 to 2.1 Intra‐run precision: 1.1%–5.7% Inter‐run accuracy: −3.1 to 2.1% Inter‐run precision: 1.9%–3.7%								
Mirvetuximab soravtansine	DM4 and S‐methyl DM4 (DM4‐Me)	8	0.100–50.0 ng/mL	5 QCs	Cumulative accuracy (%bias) DM4: −1.11% to 3.69%; DM4‐Me: −1.515 to 2.86% Cumulative precision (CV%) DM4: ≤ 6.48; DM4‐Me: ≤ 6.63 Inter‐run precision (%CV) DM4: ≤ 8.93%; DM4‐Me: ≤ 9.36%	—	No significant interference in blanks No interference of pembro‐lizumab	—	Significant carry‐over	Two‐ and tenfold dilution of 2.30 and 10.0 ng/mL, respecti‐vely	No significant matrix suppression effects	—	*(U.S. Food & Drug Administration. Drug Approval Package: ELAHERE ‐ Multi‐Discipline Review)*
Trastuzumab emtansine	Free and disulfide bound DM1 (measured as DM1‐NEM)	9	1.00–500 nM (0.737–369 ng/mL)	—	*Non‐clinical studies* Accuracy (bias%): −8.9 to 4.4 Intra‐assay precision (CV%): 6.4%–10.2% Inter‐assay precision (CV%): 2.9%–19.1% *Clinical studies* Accuracy (bias%): 0.8–14.9 Intra‐assay precision (CV%): 6.2%–9.1% Inter‐assay precision (CV%): 8.8%–17.7%	—	—	—	—	—	—	—	*(U.S. Food & Drug Administration. Drug Approval Package: KADCYLA ‐ Clinical Pharmacology and Biopharmaceutics Review(s)),* (Dere et al., 2013)
Trastuzumab emtansine	Free DM1 (as DM1‐NEM)	8	2–400 ng/mL	2.0, 5.0, 100, and 300 ng/mL	Inter‐run accuracy (%RE): −2.8 to 6.5 Inter‐run precision (CV%): 6.8 to 10.6	—	—	—	No carry over peak	—	—	—	(Jeon et al. 2023)
Trastuzumab emtansine	DM1	7	1–100 nM DM1	2, 20, and 100 nM	Accuracy (RE%): −0.7 to 5.1 Intra‐run precision: 2.0%–5.2% Inter‐run precision: 5.0%–9.5%	—	No significant interference in blank lots	No significant interference	—	—	108.2–113.8 (CV% 5.2‐10.0)	83.9%–98.9% (CV% 5.9–14.7)	(Liu et al. 2017)
Trastuzumab emtansine	DM1 (as DM1‐NEM)	6	0.5–20 ng/mL	0.5, 1.5, 7.5, and 15 ng/mL	Results based on PCI‐IS Intra‐run accuracy: 91.7% (±2.5%)–113.3% (±13.3%) Intra‐run precision (RSD%): < 9.0% Inter‐run accuracy: 89.4% (±3.5%)–113.0% (±9.6%) Inter‐run precision (RSD%): < 9.7%	—	—	—	—	—	CV%: ‐No correction: 14% ‐PCI‐IS: 3.5% ‐Normal IS: 7%	—	(Cheng et al. 2024)
Sacituzumab govitecan	SN‐38	6	5–200 ng/mL	5, 15, 75, and 150 ng/mL	Results based on PCI‐IS Intra‐run accuracy: 92.6% (±3.1%)–109.0% (±1.7%) Intra‐run precision (RSD%): < 5.0% Inter‐run accuracy: 93.9% (±2.8%)–109.7% (±5.1%) Inter‐run precision (RSD%): < 5.5	—	—	—	—	—	CV%: ‐No correction: 17% ‐PCI‐IS: 3% ‐Normal IS‐1: 20% ‐Normal IS‐2:21%	—	(Cheng et al. 2024)
Sacituzumab govitecan	SN‐38G	6	0.75–30 ng/mL	0.75, 2.25, 11.25, and 22.5 ng/mL	Results based on PCI‐IS Intra‐run accuracy: 91.7% (±2.5%)–113.3% (±13.3%) Intra‐run precision (RSD%): < 9.0% Inter‐run accuracy: 89.4% (±3.5%)–113.0% (±9.6%) Inter‐run precision (RSD%): < 9.7%	55.6 ± 8.0	—	—	—	—	CV%: ‐No correction: 6% ‐PCI‐IS: 2.5% ‐Normal IS‐1: 3% ‐Normal IS‐2: 17	—	(Cheng et al. 2024)
Total payload													
Sacituzumab govitecan	Total SN‐38	—	5.00–2500 ng/mL	Total SN‐38 LLOQ	Intra‐run accuracy: 1.5%–13.9% Intra‐run precision: 6.7%–7.5% Inter‐run accuracy: 5.8% Inter‐run precision: 8.8%	—	—	No significant interference for analyte and IS	No significant carry‐over	Two‐, five‐, and tenfold	IS‐normalized matrix factor: CV% 15 over 6 lots	SN‐38: 79.5% SN‐38‐D_3_: ongoing Hydrolysis recovery evaluation: 112.8%	*(U.S. Food & Drug Administration. Drug Approval Package: TRODELVY ‐ Multi‐Discipline Review)*
				Total SN‐38 above LLOQ	Intra‐run accuracy: −1.6% to 6.9% Intra‐run precision: 0.9%–5.1% Inter‐run accuracy: −0.6% to 4.9% Inter‐run precision: 3.0%–3.9%								

*Note:* — means that no data on this topic was available or reported.

Abbreviation: LLOQ means lower limit of quantification.

**TABLE 4:** Stability details available in the publications, grouped per ADC entity.

ADC	Compound	Bench‐top stability (hours)	Solution stability (hours)	Final extract stability (hours)	Re‐injection stability (hours)	Long‐term stability (days)	Freeze/thaw stability (cycles)	References
Intact ADC								
Tisotumab vedotin	Tisotumab vedotin	In plasma: 24 at RT and wet ice In whole blood: 2 at RT	—	—	—	374 at −80°C	5 at −20°C and −80°C	(*U.S. Food & Drug Administration. Drug Approval Package: TIVDAK—Multi‐Discipline Review*)
Amino acid–linker‐payload								
Belantamab mafodotin	Cyclic cysteine‐mcMMAF	—	—	—	—	17–266 at various Cys‐mccMMAF and ADC concentrations at −20°C 367–407 at various Cys‐mcMMAF and 150 μg/mL ADC concentration	—	(Mayer et al. 2021)
Trastuzumab emtansine	Lysine‐MCC‐DM1	3 at RT 24 at −80°C	—	24 at 4°C in autosampler	—	—	3 at −80°C	(Liu et al. 2017)
Linker‐payload								
Trastuzumab emtansine	MCC‐DM1	4 at RT	—	48 at 5°C	—	30 at −20°C and −80°C	5 at −20°C and −80°C	(Iwamoto et al. 2017)
Trastuzumab emtansine	MCC‐DM1	3 at RT 24 at −80°C	—	24 at 4°C in autosampler	—	—	3 at −80°C	(Liu et al. 2017)
Payload								
Brentuximab vedotin	Free MMAE	24 at RT	—	—	—	225 at −10°C to −30°C 390 at −60°C to −80°C	—	(*U.S. Food & Drug Administration. Drug Approval Package: ADCETRIS—Multi‐Discipline Review*)
Tisotumab vedotin	MMAE	*Method 1* 108 at 5°C *Method 2* 22 on ice or at RT	—	*Method 2* 18 at 10°C in autosampler in presence of ADC 24 at 10°C in autosampler without presence of ADC	—	*Method 2* 337 at −20°C and ‐80°C 163 at −20°C in presence of ADC 274 at −80°C in presence of ADC	*Method 2* 5 at −20°C and −70°C in or without presence of ADC in sample	(*U.S. Food & Drug Administration. Drug Approval Package: TIVDAK—Multi‐Discipline Review*)
Enfortumab vedotin	MMAE	24 on ice (plasma) 2 at RT or on ice (whole blood)	—	—	—	4 at −20°C and −70°C	5 at −20°C and −70°C	(*U.S. Food & Drug Administration. Drug Approval Package: PADCEV—Multi‐Discipline Review*)
Polatuzumab vedotin	MMAE and MMAF‐IS	—	IS stock solution of 100 mg/mL at −20°C: 8832	263 at 2°C–8°C	48 at 2°C–8°C	40 at −20°C and −70°C	5 at −20°C and −70°C	(*U.S. Food & Drug Administration. Drug Approval Package: POLIVY—Multi‐Discipline Review*)
Polatuzumab vedotin	MMAE and MMAE‐D8	6 at RT	Primary stock: 17 at RT in DMF	—	93 at RT	MMAE: 117 at −20°C MMAE‐d8: 36 at −70°C	4 at −20°C	(*U.S. Food & Drug Administration. Drug Approval Package: POLIVY*−*Multi‐Discipline Review*)
Inotuzumab ozogamicin	N‐acetyl‐γ‐calicheamicin‐DMH	*Method 1* 24 at RT (yellow light) and 2°C–8°C *Method 2* 4 at RT (yellow light) 3.18 on wet ice (yellow light)	*Method 1* Primary stock of 1.00 mg/mL in water: 6 at RT and 272 at −80°C *Method 2* Primary stock: 6.2 at RT (yellow light) and 5880 at −70°C *Methods 1 and 2* IS stock of 5.00 mg/mL: 1584 at −80°C IS working solution of 10 ng/mL in 50:50 isopropanol:water: 1	*Method 2* 56.85 at 2°C–8°C	*Method 2* 55 at 2°C–8°C	*Method 1* 297 at −20°C and −80°C *Method 2* 803 at −70°C	*Method 1* 5 at −20°C and −80°C 8 at −70°C *Method 2* 3 at −70°C	(*U.S. Food & Drug Administration. Drug Approval Package: BESPONSA—Multi‐Discipline Review*)
Inotuzumab ozogamicin	N‐acetyl‐ε‐calicheamicin	—	—	129 at 2°C–8°C	143 at 2°C–8°C	—	—	(*U.S. Food & Drug Administration. Drug Approval Package: BESPONSA—Multi‐Discipline Review*)
Trastuzumab deruxtecan	MAAA‐1181a	24 on ice in or without presence of ADC	—	—	—	579 at −20°C and −70°C without presence of ADC 112 at −20°C and 489 at −70°C in presence of ADC	5 at −20°C and −70°C in presence or absence of ADC	(*U.S. Food & Drug Administration. Drug Approval Package: ENHERTU—Multi‐Discipline Review*)
Loncastuximab tesirine	SG3199	20 at RT	—	147 at 10°C	122 at 10°C	< 64 at −20°C 348 at −70°C	5 at −70°C	(*U.S. Food & Drug Administration. Drug Approval Package: ZYNLONTA—Multi‐Discipline Review*)
Mirvetuximab soravtansine	DM4 and S‐methyl DM4 (DM4‐Me)	24 at RT in or without presence of ADC	—	—	—	1135 at −70°C	5 at −20°C and −70°C	(*U.S. Food & Drug Administration. Drug Approval Package: ELAHERE—Multi‐Discipline Review*)
Trastuzumab emtansine	DM1	4 at RT	—	29 at 4°C in autosampler (as DM1‐NEM)	—	7 or 30 at −80°C	3 at −80°C	(Jeon et al. 2023)
Trastuzumab emtansine	DM1	3 at RT 24 at −80°C	—	24 at 4°C in autosampler	—	—	3 at −80°C	(Liu et al. 2017)
Sacituzumab govitecan	Free SN‐38 and SN‐38G in presence of sacituzumab govitecan	3 on wet ice	—	71 at RT and 2°C–8°C	106	—	4 at −70°C	(*U.S. Food & Drug Administration. Drug Approval Package: TRODELVY—Multi‐Discipline Review*)
Sacituzumab govitecan	SN‐38G	6 on wet ice	—	—	—	—	3 at −20°C 4 at −70°C	(*U.S. Food & Drug Administration. Drug Approval Package: TRODELVY—Multi‐Discipline Review*)
Sacituzumab govitecan	Total SN‐38	6 at RT 21 under hydrolysis conditions	—	—	122	—	3 at −80°C	(*U.S. Food & Drug Administration. Drug Approval Package: TRODELVY—Multi‐Discipline Review*)

*Note:* — means that no data on this topic was available or reported.

Abbreviation: RT means room temperature.

### 4.1 Intact ADC

Ideally, ADC concentrations should be determined based on the intact molecule to obtain pharmacokinetic information of this molecule. However, LC–MS analysis of the intact ADC is complex because of its structure. Most ADCs have a molecular weight of around 160 kDa, multiple glycoforms, and many ionisable groups. Therefore, quantification of an intact ADC with LC–MS/MS is not possible. The intact ADC, however, can be quantified with LC–MS when using HR‐MS. One article, published by Jin et al. (2018) described an LC‐HR‐MS method for the quantification of trastuzumab emtansine in rat plasma.

#### 4.1.1 Sample Pre‐Treatment

Sample pre‐treatment of the intact ADC aims to extract the intact molecule from its matrix. This can be achieved by antibody purification, for which several approaches are described. Precipitation of the sample with ammonium sulfate results in the separation of total immunoglobulins and other serum proteins. Although this is a relatively simple method, all immunoglobulins will be present in the sample, which could cause interference and a complex LC‐HR‐MS spectrum. Therefore, more specific affinity purification methods are available using immobilized protein A, G, L or a combination of A and G. With this method, most IgG species and subclasses of IgG can be captured, including IgG1 and IgG4. Other types of affinity purification use immobilized antibodies (e.g., anti‐human IgG, anti‐idiotype, or anti‐payload antibodies), immobilized target antigens, or anti‐payload antibodies (*ThermoFisherScientific. Overview of Affinity Purification*; *WuXiAppTec. Antibody‐Drug Conjugate (ADC) Bioanalysis Strategies Based on LC‐MS*). For the analysis of intact ADCs, anti‐payload antibodies could be more selective. However, when the complete DAR profile is to be quantified, the naked antibody (with DAR 0) is not determined with this type of immunity purification. One of the challenges with LC‐HR‐MS ADC analysis, is the complexity of mass spectra that are obtained. To simplify the complex mass spectra of the ADCs, a de‐*N*‐glycosylation step could be performed as part of sample pre‐treatment. This prevents interference as of overlapping mass‐to‐charge ions for *N*‐linked glycosylation and linker‐payload attachment (Friese et al. 2018).

In the published method for trastuzumab emtansine, immuno‐affinity purification was performed using biotinylated goat anti‐human IgG bound to streptavidin magnetic beads (Jin et al. 2018), resulting in the complete DAR profile of trastuzumab emtansine. After addition of the beads to the rat plasma sample, incubation, washing, and elution, the final extract was neutralized and analyzed with LC‐HR‐MS. No stable isotopically labeled mAb was used as internal standard, as this complicated the mass spectrum because of insufficient chromatographic separation. No de‐*N*‐glycosylation step was part of the sample pre‐treatment (Jin et al. 2018).

#### 4.1.2 Validation Criteria

Quantification of trastuzumab emtansine was assessed for two types of HR‐MS data processing methods. The first method employed quantification using extracted ion chromatograms (XICs). The 53+ charge state of glycoform G0F‐1/G1F‐1 of DAR3 trastuzumab emtansine (m/z of 2851.7697 and XIC width of 5 mDa) was best for quantification of trastuzumab emtansine. The concentration range of 5–100 μg/mL trastuzumab emtansine was linear with a recovery of 70%. The second method described quantification based on deconvoluted mass peak areas of five specific trastuzumab emtansine protein forms. These included glycoforms G0F‐2, G0F‐1/G1F‐1, G0F‐1/G2F‐1 or G1F‐2 of trastuzumab emtansine with DAR3, the total peak area of trastuzumab emtansine DAR3, and total peak areas of all DARs. For all five protein forms, the range of 5–100 μg/mL trastuzumab emtansine was linear and accurate and precise (Jin et al. 2018).

For the quantification of ADCs with LC‐HR‐MS, quantification using deconvoluted mass peak areas of all DARs instead of using one specific DAR is advised. Since DARs change over time due to biotransformation or drug release in vivo, choosing only one DAR could lead to an under‐ or overestimation of the ADC concentration (Jin et al. 2018).

#### 4.1.3 Stability

No stability experiments were performed. For correct quantification of the intact ADC, stability data should be obtained to ensure no payload is released during sample processing, bench‐top, or long‐term storage. Specifically for intact ADC analysis, linker‐payload present at the surface of the ADC, could be fragile and susceptible for in‐source fragmentation. This could result in loss of linker‐payload, thereby, inadequately determining the intact ADC concentration (Friese et al. 2018).

#### 4.1.4 Clinical Applicability

Before the method can be clinically applied, the assay range should be optimized to ensure sensitive measurements. The validated range could be suitable for peak level measurements. As part of registration studies, peak levels of 83.4 (±16.5) μg/mL and 72.6 (±24.3) μg/mL trastuzumab emtansine were found (*U.S. Food & Drug Administration. Drug Approval Package: KADCYLA—Clinical Pharmacology and Biopharmaceutics Review(s)*). The reported plasma half‐life is approximately 4 days, resulting in a trough level after 1 cycle of below the lower limit of quantification (LLOQ) of this method (*U.S. Food & Drug Administration. Drug Approval Package: KADCYLA—Clinical Pharmacology and Biopharmaceutics Review(s)*). Since no accumulation was observed for this drug (*U.S. Food & Drug Administration. Drug Approval Package: KADCYLA—Clinical Pharmacology and Biopharmaceutics Review(s)*), the assay would not be sensitive enough for accurate trough level measurements of trastuzumab emtansine.

### 4.2 Total Antibody

With a total antibody assay, the total concentration of the monoclonal antibody regardless of payload conjugation is measured. This concentration is often assessed to study the efficacy as well as on‐target toxicity (Mahmood 2021). One LC–MS/MS method for the determination of the total antibody concentration of an ADC was described, which was for trastuzumab emtansine (Iwamoto et al. 2017).

#### 4.2.1 Sample Pre‐Treatment

Like the measurement of intact ADCs, the measurement of intact antibodies with LC–MS is complex. Therefore, sample pre‐treatment aims to generate peptides that are specific and selective for the antibody to measure. These are called signature peptides and generally have a molecular weight of 1000–3000 Da, which can sensitively be measured with LC–MS/MS as a surrogate for the monoclonal antibody. Multiple methods for the quantification of monoclonal antibodies with LC–MS/MS have already been published (de Jong et al. 2022; Li et al. 2024; Schokker et al. 2020). Sample pre‐treatment of spiked plasma samples with trastuzumab emtansine was performed using nano‐surface and molecular‐orientation limited (nSMOL) proteolysis. Using nSMOL only peptides of the antigen binding region (Fab) of the monoclonal antibody are formed. The selected signature peptide for trastuzumab quantification was IYPTNGYTR. With the use of a P14R synthetic peptide as internal standard, the trastuzumab concentration in trastuzumab emtansine samples could be determined with LC–MS/MS (Iwamoto et al. 2017).

#### 4.2.2 Validation Criteria

The assay was validated according to the Guideline on Bioanalytical Method Validation in Pharmaceutical Development of the Japanese Pharmaceuticals and Medical Devices Agency. However, only the range, mean accuracy, and overall precision results were reported (Iwamoto et al. 2017).

#### 4.2.3 Stability

No results of stability experiments were reported in this assay. Although stability experiments are important to ensure stability of the monoclonal antibody during sample pre‐treatment and storage, the stability of ADC is less important, as the total antibody concentration is determined regardless of conjugated payload.

#### 4.2.4 Clinical Applicability

The validated range for quantification was 0.061–250 μg/mL trastuzumab emtansine, which is a wide range suitable for clinical application. Twenty‐one patient samples were measured as part of clinical application, resulting in a mean trastuzumab concentration of approximately 10 μg/mL with unknown time after dose (Iwamoto et al. 2017). The total antibody concentrations were similar as in registration studies according to the authors and fell within the validated range.

### 4.3 Amino Acid–Linker‐Payload

A residual amino acid covalently attached to a linker‐payload can be the result of proteolysis of an ADC with a non‐cleavable linker. As the potency of the payload is not necessarily impaired due to the addition of an amino acid and linker (McCombs and Owen 2015), measuring this ADC entity is useful since it could contribute to efficacy or toxicity.

#### 4.3.1 Sample Pre‐Treatment

Sample pre‐treatment aims to obtain the amino acid–linker‐payload entity without additional introduction of this moiety derived from the intact ADC during this process. Two LC–MS/MS methods were available describing the extraction and LC–MS/MS method for Lys‐MCC‐DM1 and Cys‐mcMMAF, originating from trastuzumab emtansine and belantamab mafodotin, respectively. Protein precipitation (PPT) in an acidic environment and solid phase extraction (SPE) were used for sample pre‐treatment (Liu et al. 2017; Mayer et al. 2021). The difference between PPT and SPE is the presence of the ADC in the sample. Therefore, the potential influence of intact ADC still present in the sample should be investigated. For the determination of Cys‐mcMMAF, SPE and acidic PPT were compared, and resulted in an overestimation of the amino acid–linker‐payload concentration with SPE compared to PPT (Mayer et al. 2021). Assuming that the intact ADC molecule is present in patient samples, is could be a downside for this technique as sample pre‐treatment method.

The FDA Clinical Pharmacology and Biopharmaceutics Review did not describe any sample pre‐treatment details and is, therefore, not shown in Table 2 (*U.S. Food & Drug Administration. Drug Approval Package: KADCYLA—Clinical Pharmacology and Biopharmaceutics Review(s)*).

#### 4.3.2 Validation Criteria

For all amino acid–linker‐payload LC–MS/MS assays, accuracy and precision data were reported (Liu et al. 2017; Mayer et al. 2021; *U.S. Food & Drug Administration. Drug Approval Package: KADCYLA—Clinical Pharmacology and Biopharmaceutics Review(s)*). Interestingly, accuracy and precision of Cys‐mcMMAF was assessed in presence of belantamab mafodotin, resulting in accurate and precise results using acidic PPT as sample pre‐treatment and a 1.5‐fold increased Cys‐mcMMAF concentration using SPE (Mayer et al. 2021). The assay published by Liu et al. (2017) reported more validation results, including selectivity, matrix effect, and recovery results (Liu et al. 2017). The FDA Clinical Pharmacology and Biopharmaceutics Review did not include any other results on validation experiments (*U.S. Food & Drug Administration. Drug Approval Package: KADCYLA—Clinical Pharmacology and Biopharmaceutics Review(s)*).

#### 4.3.3 Stability

Most important issue for the determination of the amino acid–linker‐payload is the stability of the intact ADC. Only for Cys‐mcMMAF long‐term stability was assessed in presence of the ADC (Mayer et al. 2021). Other stability experiments were not performed. For Lys‐MCC‐DM1, the stability was tested in absence of the ADC at bench‐top conditions, in the final extract, and after three freeze/thaw cycles (Liu et al. 2017). No stability results were shown by the FDA Clinical Pharmacology and Biopharmaceutics Review (*U.S. Food & Drug Administration. Drug Approval Package: KADCYLA—Clinical Pharmacology and Biopharmaceutics Review(s)*).

#### 4.3.4 Clinical Applicability

The described ranges for the determination of Lys‐MCC‐DM1 were between 1.00 and 500 nM, corresponding with 1.10 and 552 ng/mL but were measured in a different matrix, plasma, and cell suspension (Liu et al. 2017; *U.S. Food & Drug Administration. Drug Approval Package: KADCYLA—Clinical Pharmacology and Biopharmaceutics Review(s)*). According to exploratory analyses by Girish et al. (2012) for registration purpose, the mean Lys‐MCC‐DM1 concentration in plasma was 1.35 ng/mL (range 1.25–1.45 ng/mL) 1 h after infusion of 3.6 mg/kg trastuzumab emtansine in the first cycle and were comparable with the concentrations 1 hour after infusion in Cycle 3 (Girish et al. 2012). Post‐dose levels could be determined, although already being close to the LLOQ. For the measurement of pre‐dose levels, however, the assay should be optimized.

Cys‐mcMMAF could be determined between 50 and 10,000 pg/mL (Mayer et al. 2021). As part of the registration study DREAMM‐2, Rathi et al. (2021) calculated the maximum concentration and average concentration of Cys‐mcMMAF based on patient samples. Around 25% of the samples were below the LLOQ. Of the other 75%, the average concentration of Cys‐mcMMAF was 217 pg/mL and the maximum concentration was 917 pg/mL after a dose of 2.5 mg/kg belantamab mafodotin (Rathi et al. 2021). Most levels were within the validated range published by Mayer et al. (2021), however method optimization would be preferred for good clinical application of this method.

### 4.4 Linker‐Payload

Hydrolysis of the linker‐payload from the ADC or the amino acid–linker‐payload entity can result in the formation of the linker‐payload entity (Cherifi et al. 2024).

#### 4.4.1 Sample Pre‐Treatment

Four publications described the quantification of linker‐payload MCC‐DM1 with LC–MS/MS (Cheng et al. 2024; Iwamoto et al. 2017; Liu et al. 2017; *U.S. Food & Drug Administration. Drug Approval Package: KADCYLA—Clinical Pharmacology and Biopharmaceutics Review(s)*). Three of these publications did include details on sample pre‐treatment, which was performed using liquid–liquid extraction (LLE) or PPT (Cheng et al. 2024; Iwamoto et al. 2017; Liu et al. 2017). LLE requires more handling than PPT, but with both sample pre‐treatment methods the intact ADC is removed from the sample and, therefore, the risk of ex vivo linker‐payload release is minimal. Still, it is advised to investigate the influence of intact ADC presence in the sample.

#### 4.4.2 Validation Criteria

The FDA Clinical Pharmacology and Biopharmaceutics review did not report any validation results, except for the range (*U.S. Food & Drug Administration. Drug Approval Package: KADCYLA—Clinical Pharmacology and Biopharmaceutics Review(s)*). The three other publications included the range, and accuracy and precision data. Two of these publications results on selectivity, carry‐over, matrix effect, and recovery (Cheng et al. 2024; Iwamoto et al. 2017; Liu et al. 2017).

#### 4.4.3 Stability

In two publications, the stability of MCC‐DM1 was assessed in absence of trastuzumab emtansine at bench‐top, in final extract, at long‐term storage, and after at least three freeze/thaw cycles (Iwamoto et al. 2017; Liu et al. 2017).

#### 4.4.4 Clinical Applicability

The ranges for the quantification of MCC‐DM1 mostly overlap and cover the range of 0.391–488 ng/mL MCC‐DM1 (Cheng et al. 2024; Iwamoto et al. 2017; Liu et al. 2017; *U.S. Food & Drug Administration. Drug Approval Package: KADCYLA—Clinical Pharmacology and Biopharmaceutics Review(s)*). Girish et al. (2012) found a mean MCC‐DM1 concentration of 34.4 ng/mL in a range of 6.47–122 ng/mL 1 h after infusion of 3.6 mg/kg trastuzumab emtansine in Cycle 1 (Girish et al. 2012). Concentrations were not significantly different in Cycle 3 (Girish et al. 2012). Most ranges for MCC‐DM1 quantification would be suitable for clinical application of peak level measurements. However, for the measurement of trough levels, optimization of the method would be advised.

### 4.5 Payload

#### 4.5.1 Conjugated Payload

Measuring the conjugated payload concentration reflects the amount of active cytotoxic molecules bound to the monoclonal antibody and, therefore, the bioactivity of the ADC (see Figure 1) (Qin and Gong 2022).

Three LC–MS/MS methods were published for the determination of conjugated payload, which were used as a surrogate for the intact ADC concentration.

##### 4.5.1.1 Sample Pre‐Treatment

To determine the conjugated payload concentration, ADCs should be isolated from the sample ensuring that no free payload is present. For the extraction of polatuzumab vedotin and trastuzumab emtansine, immuno‐affinity capture was used (*U.S. Food & Drug Administration. Drug Approval Package: POLIVY—Multi‐Discipline Review*; Yuan et al. 2024), though the specific type of immune‐affinity capture was not detailed. This approach effectively washes away free payload and other molecules present in the sample. The extraction of inotuzumab ozogamicin was performed using LLE with methyl tert‐butyl ether (MTBE) and water. The aqueous phase contained inotuzumab ozogamicin and the MTBE phase contained the unconjugated payload fraction (*U.S. Food & Drug Administration. Drug Approval Package: BESPONSA—Multi‐Discipline Review*). Once the ADC is captured, the next step is to release the conjugated payload. Payload release depends on the properties of the linker. Polatuzumab vedotin has a cleavable protease‐sensitive linker, for which papain was used to cleave the dipeptide linker which resulted in the release of MMAE (*U.S. Food & Drug Administration. Drug Approval Package: POLIVY—Multi‐Discipline Review*). Inotuzumab ozogamicin has an acid‐labile linker, which is hydrolysed at low pH. In vivo, after hydrolysis, intracellular glutathione reduces the disulfide bond to release the payload. In the sample pre‐treatment of inotuzumab ozogamicin, the payload was directly released by the reduction of the disulfide bridge with dithiotreitol, without requiring an acidic environment for the cleavage of the acid‐labile hydrazine linker (*U.S. Food & Drug Administration. Drug Approval Package: BESPONSA—Multi‐Discipline Review*). Trastuzumab emtansine contains a non‐cleavable MCC linker, for which payload release depends on lysosomal proteolytic degradation. After capturing trastuzumab emtansine, the heavy and light chains were reduced with dithiotreitol followed by deglycosylation. Collision‐induced dissociation (CID) was then used to release the payload from the heavy and light chains as part of a digestion‐free middle‐down mass spectrometry (DF‐MDMS) method (Yuan et al. 2024). Quantification was only successful for the light chain with one and two conjugated payloads, possibly due to heterogeneity of payload conjugation on the heavy chain. This method for measuring the conjugated payload may be more suitable for ADCs with cysteine‐conjugated payloads, as these generally exhibit less heterogeneity compared to lysine‐conjugated payloads.

##### 4.5.1.2 Validation Criteria

For all three LC–MS/MS assays, the range, accuracy, and precision results were reported (*U.S. Food & Drug Administration. Drug Approval Package: BESPONSA—Multi‐Discipline Review*; *U.S. Food & Drug Administration. Drug Approval Package: POLIVY—Multi‐Discipline Review*; Yuan et al. 2024). For inotuzumab ozogamicin and polatuzumab vedotin, selectivity, dilution integrity, and recovery were additionally described. For trastuzumab emtansine, the signal‐to‐noise for the LLOQ was additionally reported.

##### 4.5.1.3 Stability

Although the conjugated payload was determined with the described LC–MS/MS assays, stability was only assessed for the payload in absence of the ADC. This is surprising since stability of the ADC is important to accurately measure the conjugated payload concentration (*U.S. Food & Drug Administration. Drug Approval Package: BESPONSA—Multi‐Discipline Review*; *U.S. Food & Drug Administration. Drug Approval Package: POLIVY—Multi‐Discipline Review*; Yuan et al. 2024). Stability of the ADC itself during sample pre‐treatment is another important consideration. Due to light sensitivity of sacituzumab govitecan and inotuzumab ozogamicin, sample pre‐treatment should be conducted under yellow light (*bc Cancer Drug Manual. DRUG NAME: Inotuzumab ozogamicin*; *Gilead Medical Information. Trodelvy Storage and Stability (Reconstituted and Diluted)*). However, for sacituzumab govitecan, no data was available of stability testing under such conditions. For inotuzumab ozogamicin, both bench‐top and solution stability were carried out under yellow light to test appropriate sample pre‐treatment conditions (*U.S. Food & Drug Administration. Drug Approval Package: BESPONSA—Multi‐Discipline Review*).

##### 4.5.1.4 Clinical Applicability

Two out of three LC–MS/MS assays are suitable for clinical application (*U.S. Food & Drug Administration. Drug Approval Package: BESPONSA—Multi‐Discipline Review*; *U.S. Food & Drug Administration. Drug Approval Package: POLIVY—Multi‐Discipline Review*). For polatuzumab vedotin, the final extract containing MMAE was measured in a concentration range of 0.5–50.0 nM MMAE, corresponding with 0.359–35.9 ng/mL. This range is sensitive enough for trough level PK measurements, since only 215 out of 4215 (4.5%) of the first post‐dose samples were below the LLOQ of this range (Lu et al. 2020). The range for *N*‐acetyl‐ε‐calicheamicin was 1.00–500 ng/mL. PK simulations performed for inotuzumab ozogamicin resulted in a concentration ranging between 10 and 500 ng/mL at the end of Cycle 3 in adults and children (Wu et al. 2024). As these concentrations are within the assay range, could be clinically applied.

The assay quantifying conjugated DM1 to trastuzumab emtansine needs optimization for clinical application. Only light chains could be quantified in ranges of 3000–90,000 and 9000–90,000 ng/mL with one or two conjugated cytotoxic molecules in mouse plasma, respectively (Yuan et al. 2024).

#### 4.5.2 Unconjugated Payload

Unconjugated payload refers to the amount of payload not bound to the ADC (see Figure 1). This includes free payload present in plasma or serum, as well as cytotoxic molecules that have formed dimers or adducts with molecules like albumin, glutathione, or cysteine. Determination of unconjugated payload is important, as premature release of the payload can lead to off‐target toxicities such as hepatotoxicity and hematological toxicity (Fu et al. 2022). LC–MS/MS methods for measuring unconjugated payload concentrations have been described in 16 publications.

##### 4.5.2.1 Sample Pre‐Treatment

Separation of conjugated and unconjugated payload during sample pre‐treatment is a crucial first step in the bioanalysis of unconjugated payload. Before LC–MS/MS analysis, three sample pre‐treatment methods were typically described for the extraction of the unconjugated payload fraction: SPE, supported liquid extraction (SLE), and PPT either alone or combined with online SPE. These methods proved to be effective to measure payload, as most cytotoxic molecules used as payload are hydrophobic in nature. Specific details about sample pre‐treatment were often not included in the descriptions of the methods, which are crucial to distinguish whether the free payload concentration only or also payload dimers or adducts were measured. The chemical properties of the dimers and adducts in combination with the chemicals used during sample pre‐treatment will decide what concentration is measured. For the determination of the unconjugated payload of trastuzumab emtansine, sample pre‐treatment was briefly described. Since DM1 contains a sulfide that could react with other sulfide containing molecules, DM1 was measured after reduction and subsequent derivatization with *N*‐ethylmaleimide (NEM) (Dere et al. 2013; Jeon et al. 2023; *U.S. Food & Drug Administration. Drug Approval Package: KADCYLA—Clinical Pharmacology and Biopharmaceutics Review(s)*), thereby measuring free as well as unconjugated payload. For the determination of polatuzumab vedotin, PPT was used. However, based on its cysteine conjugation, adduct formation of the linker‐payload entity with albumin could take place. With PPT, this entity is removed and, thereby, not measured with LC–MS/MS. Details on chemicals or reagents used for sample pre‐treatment are also critical to ensure stability of the unconjugated payload and prevent unintended payload release from the ADC. For example, inotuzumab ozogamicin contains an acid sensitive linker, which can be cleaved in acidic conditions. pH control during sample pre‐treatment is essential to prevent premature payload release during sample processing, thereby increasing the unconjugated payload concentration.

##### 4.5.2.2 Validation Criteria

For all published LC–MS/MS assays measuring unconjugated payload, key validation parameters, calibration range, accuracy, and precision were consistently reported. These parameters are most important, as they determine whether patient samples can be reliably quantified within clinically relevant concentration ranges. Other validation criteria, such as LLOQ signal‐to‐noise ratio, selectivity and specificity, carry‐over, matrix effect, and recovery were less consistently reported. These parameters were more frequently detailed in peer‐reviewed publications than in FDA reports.

##### 4.5.2.3 Stability

For the accurate determination of the unconjugated payload concentration, ensuring stability during storage and sample pre‐treatment is essential. To accurately quantify concentrations reflecting the situation in patients at the moment of sampling, stability assessments should be conducted in the presence of the intact ADC, as its presence can influence the measured levels of unconjugated payload due to potential degradation or payload release over time.

Short‐term stability in presence of the ADC was evaluated for mirvetuximab soravtansine, while long‐term stability in presence of the ADC was evaluated for tisotumab vedotin and trastuzumab deruxtecan. These results showed that the stability of the unconjugated payload was generally shorter in presence of the ADC than in its absence (*U.S. Food & Drug Administration. Drug Approval Package: ELAHERE—Multi‐Discipline Review*; Yin et al. 2021), highlighting the possibility of payload release or degradation of the conjugated form in the patient sample over time. For enfortumab vedotin, bench‐top stability of MMAE in whole blood and plasma was investigated (*U.S. Food & Drug Administration. Drug Approval Package: PADCEV—Multi‐Discipline Review*). MMAE was less stable in whole blood, suggesting that patient sample processing should take place within 2 hours to preserve the real patient condition. However, it was not reported whether this instability results from MMAE degradation itself or from release of the ADC (*U.S. Food & Drug Administration. Drug Approval Package: PADCEV—Multi‐Discipline Review*).

Stress testing can be valuable in evaluating ADC degradation post‐sampling. For SN‐38, attached with an acid‐labile linker to form sacituzumab govitecan, hydrolytic conditions were tested for bench‐top stability in the determination of total SN‐38. Interestingly, potential hydrolysis and SN‐38 release over time in the sample was not determined (*U.S. Food & Drug Administration. Drug Approval Package: TRODELVY—Multi‐Discipline Review*).

##### 4.5.2.4 Clinical Applicability

The measurable unconjugated payload fraction depends on multiple factors, including linker stability, cytotoxin clearance, and the DAR at a given time point. Generally, high assay sensitivity is required due to low concentrations of unconjugated payload in the circulation. Predicted or measured mean trough or peak concentrations of unconjugated payload concentrations were available for all ADCs for which an unconjugated payload LC–MS/MS assay was described. Among these, a few methods demonstrated sufficient sensitivity to accurately quantify trough concentrations. For MMAE, assays with a lower limit of quantitation (LLOQ) of at least 35.9 pg/mL were able to quantify at least 90% of trough‐level samples, making all described LC–MS/MS assays suitable for clinical application (*U.S. Food & Drug Administration. Drug Approval Package: ADCETRIS—Multi‐Discipline Review*; *U.S. Food & Drug Administration. Drug Approval Package: PADCEV—Multi‐Discipline Review*; *U.S. Food & Drug Administration. Drug Approval Package: POLIVY—Multi‐Discipline Review*; *U.S. Food & Drug Administration. Drug Approval Package: TIVDAK—Multi‐Discipline Review*). In the case of SN‐38 and SN‐38G, released from sacituzumab govitecan, median concentrations of 3.00 ng/mL were measured (Sathe et al. 2024). These levels could be quantified using methods with a LLOQ of 1.00 ng/mL. Similarly, for MAAA‐1181a, the payload released by trastuzumab deruxtecan, a plasma concentration profile ranging from 0.1 to 1.0 ng/mL was observed following 5.4 mg/kg trastuzumab deruxtecan administration (Yin et al. 2021). These concentrations could be measured accurately using the method developed by the FDA (Yin et al. 2021), while the method described by Cheng et al. (2024) lacked sufficient sensitivity for this application (Cheng et al. 2024).

For the remaining ADCs, the published LC–MS/MS methods were not sensitive enough to quantify trough concentrations. Optimization for better sensitivity may be required to ensure clinical applicability. However, if the ADC is highly stable and only very low unconjugated payload levels are expected, such optimizations might be unnecessary, as these minimal concentrations are unlikely to significantly contribute to toxicity.

#### 4.5.3 Total Payload

The measurement of total payload can useful to calculate the conjugated payload concentration with the determination of the unconjugated payload concentration. The only LC–MS/MS assay quantifying total payload was described for sacituzumab govitecan.

##### 4.5.3.1 Sample Pre‐Treatment

To determine the total payload concentration, the conjugated payload should be released during sample pretreatment. For sacituzumab govitecan, total payload was measured after hydrolysis of the linker, resulting in free SN‐38 as well as acid‐dissociated SN‐38 present in the sample. Subsequently, PPT was performed to extract the total amount of SN‐38 (*U.S. Food & Drug Administration. Drug Approval Package: TRODELVY—Multi‐Discipline Review*). Details about used reagents in sample pre‐treatment were not provided.

##### 4.5.3.2 Validation Criteria

The results of the following validation experiments were reported, including range, accuracy and precision, selectivity, carry‐over, dilution integrity, matrix effect, and recovery. Since during sample pre‐treatment SN‐38 is dissociated from the antibody, SN‐38 hydrolysis efficiency was also assessed (*U.S. Food & Drug Administration. Drug Approval Package: TRODELVY—Multi‐Discipline Review*).

##### 4.5.3.3 Stability

Results on stability experiments were sparsely reported and included only benchtop stability at room temperature and at hydrolysis conditions, re‐injection stability, and freeze/thaw stability of SN‐38 (*U.S. Food & Drug Administration. Drug Approval Package: TRODELVY—Multi‐Discipline Review*). It was not described whether the ADC was present or not in the sample. For the measurement of the total payload concentration, this is not very important as conjugated as well as unconjugated payload will be determined.

##### 4.5.3.4 Clinical Applicability

The assay was developed to measure SN‐38 in a range of 5.00–2500 ng/mL (*U.S. Food & Drug Administration. Drug Approval Package: TRODELVY—Multi‐Discipline Review*). Total SN‐38 concentrations were determined by Sathe et al. (2024) but the exact values were not reported. The LLOQ for the total SN‐38 was five times higher than for unconjugated SN‐38 measurements, which would be appropriate for clinical application since the total SN‐38 concentration will be higher than the median range of unconjugated SN‐38, which was 3–100 ng/mL.

## 5 Discussion and Future Perspectives

In this review, we provided an overview of published LC–MS methods for quantifying of all entities of an ADC, focusing on ADCs that are registered or under evaluation by the EMA. Based on the reviewed literature, we conclude that the majority of LC–MS methods were described in the FDA Multi‐Disciplinary Reviews or the FDA Clinical Pharmacology Biopharmaceutics Review(s). Bioanalytical information in these reports only briefly discuss the sample pre‐treatment procedure and often lack chromatography or mass spectrometry details. Validation criteria that were assessed and reported aligned with those required for determination of pharmacokinetic parameters investigated in (non‐)clinical registration trials but often do not include all validation criteria specified by the FDA or EMA Guidelines on bioanalytical method validation. In contrast, LC–MS assays that have been developed, validated, and published as full manuscripts, did provide more details about sample pre‐treatment, chromatography, mass spectrometry, and assessed validation criteria. Except for one LC–MS assay, the assessed validation criteria of the described LC–MS assays met the FDA Guidelines for Bioanalytical Method Validation. For each ADC entity, at least one quantitative LC–MS method was available. However, overall, the number of assays and the provided information was scarce. Most available LC–MS/MS methods described the quantification of linker‐payload or payload concentrations, which is not surprising, given that LC–MS/MS methods are more common for small‐molecule analysis (Qin and Gong 2022). Notably, LC–MS methods for each ADC entity of trastuzumab emtansine have been published.

Due to the complexity of ADC molecules and the various entities that may be present after administration, better understanding of their clinical pharmacology is essential to improve efficacy and toxicity of these drugs. This can be achieved through the development of new bioanalytical assays able to differentiate between these entities. As demonstrated in this review, quantification of the different ADC entities is feasible with LC–MS. When developing LC–MS methods in the future, several important factors should be considered.

Quantifying intact ADCs by LC–MS requires careful optimization due to the complexity of the molecule and the low sensitivity of HR‐MS, which creates challenges for clinical application. However, pharmacokinetic data of ADCs can also be obtained by analyzing the other ADC entities, including total antibody concentration, (amino acid) linker‐payload concentration, the conjugated payload concentration, and the unconjugated payload concentration. For all these entities, it should be established that the measurements represent the concentration in the sample at the moment of sampling. Key is the potential payload release during sample pre‐treatment that should be prevented, necessitating thorough evaluation of linker stability under these conditions. Alongside sample pre‐treatment considerations, the stability of each ADC entity should be extensively assessed both in presence and absence of the ADC to ensure reliable measurements after sample storage. Once developed, the LC–MS method should be validated following current EMA or FDA guidelines. This validation guarantees accuracy, precision, and reliability for quantifying concentrations in patient samples. After successful validation, the method could be applied to clinical samples to evaluate its clinical applicability and contribute to pharmacokinetic data on ADCs.

## 6 Conclusion

This review provides an overview of published LC–MS methods for the quantification of all entities of an ADC, based on the ADCs that are registered or under evaluation by the EMA. For accurate determination of in vivo pharmacokinetics of ADCs, we consider LC–MS most suited as bioanalytical method. This review could serve as a starting point for LC–MS method development for all ADC entities. Here, two main forms of the ADC are important for pharmacokinetic profiling to investigate efficacy as well as toxicity relationships. First, the intact ADC concentration could be measured either directly by LC‐HR‐MS, which is challenging, or by combining the quantification of several ADC entities. The total antibody concentration and conjugated payload concentration can be used to calculate the ADC concentration based on the average DAR of the formulated product of the ADC. Ideally, the average DAR of the ADC in vivo would be used for this calculation as DAR changes over time. This requires ADC characterization in vivo per patient sample with LC‐HR‐MS (Xu et al. 2011). Second, the unconjugated payload concentration is of interest as this could be mostly related to toxicity as of premature release of the cytotoxic payload in the systemic circulation.

## Funding

The authors have nothing to report.

## Disclosure

Neeltje Steeghs has financial interests: Boehringer Ingelheim (Advisory Board, Institutional), Bristol‐Myers Squibb (Advisory Board, Institutional), Ellipses Pharma (Advisory Board, Institutional), GlaxoSmithKline (Advisory Board, Personal), Incyte (Advisory Board, Personal), Abbvie (Local PI, Institutional), Actuate Therapeutics (Local PI, Institutional), Amgen (Local PI, Institutional), Anaveon (Local PI, Institutional), AstraZeneca (Local PI, Institutional), AstraZeneca (Research Grant, Institutional), Bayer (Local PI, Institutional), Blueprint Medicines (Local PI, Institutional), Blueprint Medicines (Research Grant, Institutional), Boehringer Ingelheim (Local PI, Institutional), Bristol‐Myers Squibb (Local PI, Institutional), CellCentric (Local PI, Institutional), Cogent Biosciences (Local PI, Institutional), Crescendo Biologics (Local PI, Institutional), Daiichi Sayko (Local PI, Institutional), Deciphera (Local PI, Institutional), Deciphera (Research Grant, Institutional), Exelixis (Local PI, Institutional), Genentech (Local PI, Institutional), GlaxoSmithKline (Local PI, Institutional), GlaxoSmithKline (Research Grant, Institutional), Iambic (Local PI, Institutional), IDRx (Local PI, Institutional), Immunocore (Local PI, Institutional), Incyte (Local PI, Institutional), Janssen (Local PI, Institutional), Kling Biotherapeutics (Local PI, Institutional), Lixte (Research Grant, Institutional), Merck (Local PI, Institutional), Merck (Research Grant, Institutional), Merck Sharp & Dohme (Local PI, Institutional), Merus (Local PI, Institutional), Molecular Partners (Local PI, Institutional), Novartis (Local PI, Institutional), Novartis (Research Grant, Institutional), Pfizer (Local PI, Institutional), Pfizer (Research Grant, Institutional), Revolution Medicin (Local PI, Institutional), Roche (Local PI, Institutional), Roche (Research Grant, Institutional), Sanofi (Local PI, Institutional), Zentalis (Research Grant, Institutional). None of these interests were related to the submitted manuscript.

Jos H. Beijnen is co‐founder of a patent on oral taxane formulations and is a (part‐time) employee and (indirect) shareholder of Modra Pharmaceuticals BV, a small spin‐out company of the Netherlands Cancer Institute (Amsterdam, the Netherlands) developing oral taxane treatment. This is not related to the submitted manuscript.

The other authors declare that they have no known competing financial interests or personal relationships that could have appeared to influence the work reported in this paper.

## Data Availability

Data sharing is not applicable to this article as no datasets were generated or analyzed during the current study.
